# Analysis of Identification Method for Bacterial Species and Antibiotic Resistance Genes Using Optical Data From DNA Oligomers

**DOI:** 10.3389/fmicb.2020.00257

**Published:** 2020-02-20

**Authors:** Ryan L. Wood, Tanner Jensen, Cindi Wadsworth, Mark Clement, Prashant Nagpal, William G. Pitt

**Affiliations:** ^1^Chemical Engineering, Brigham Young University, Provo, UT, United States; ^2^Computer Science, Brigham Young University, Provo, UT, United States; ^3^Chemical and Biological Engineering, University of Colorado Boulder, Boulder, CO, United States

**Keywords:** antibiotic resistance, machine learning, DNA sequencing, Raman spectroscopy, biomedical diagnostic

## Abstract

Bacterial antibiotic resistance is becoming a significant health threat, and rapid identification of antibiotic-resistant bacteria is essential to save lives and reduce the spread of antibiotic resistance. This paper analyzes the ability of machine learning algorithms (MLAs) to process data from a novel spectroscopic diagnostic device to identify antibiotic-resistant genes and bacterial species by comparison to available bacterial DNA sequences. Simulation results show that the algorithms attain from 92% accuracy (for genes) up to 99% accuracy (for species). This novel approach identifies genes and species by optically reading the percentage of A, C, G, T bases in 1000s of short 10-base DNA oligomers instead of relying on conventional DNA sequencing in which the sequence of bases in long oligomers provides genetic information. The identification algorithms are robust in the presence of simulated random genetic mutations and simulated random experimental errors. Thus, these algorithms can be used to identify bacterial species, to reveal antibiotic resistance genes, and to perform other genomic analyses. Some MLAs evaluated here are shown to be better than others at accurate gene identification and avoidance of false negative identification of antibiotic resistance.

## Introduction

Novel DNA sequencing technologies have proliferated over the past two decades. Continual improvements in “next-generation sequencing” (NGS) and “third-generation sequencing” (TGS) have increased the fidelity and rate of sequencing, but it still takes hours or days to obtain complete sequences (van Dijk et al., 2018). Sequencing plays an essential role in biological classification, cell biology, forensic analysis, and gene manipulation for medical and research purposes. Furthermore, there are some diagnostic applications in which very rapid identification of a particular gene or genetic species becomes essential, while identification of all genes is not necessary. For example, in patients with septic shock from bacterial infections, identification of antibiotic-resistance genes is essential because the mortality rate increases 7.6% per hour of delay in administering correct antibiotics (Kumar et al., 2006). Unfortunately, it takes more than 24 h to grow up the bacteria recovered from the blood of an infected patient, identify the species, and then determine to which antibiotics the organism is resistant, leading to a very high mortality rate for such infections (Kumar et al., 2009). Carbapenem resistance is one of the most concerning antibiotic resistances, as infections with carbapenem-resistant bacteria have a 48% mortality rate (Patel et al., 2008) caused in part by the good reluctance of physicians to initially prescribe carbapenem antibiotics without verifying resistance because of the severe side effects of carbapenems. The attending clinician wants to know the bacterial species and needs to know any resistance to antibiotics, with confidence that the diagnostic technique has a very low error rate of false negatives. As genome and plasmid sequencing can identify the species and previously identified resistance genes, it would be tremendously useful to perform bacterial sequencing in an hour or less. However, current and proposed NGS and TGS techniques still require much more time.

Herein we present a novel approach that is useful when the diagnostic objective is to rapidly identify the species of bacteria or the presence of an antibiotic resistance gene in the bacteria. Our approach employs a genomic analysis technique that has some data compression and data loss, but compensates by very rapid analysis of very short reads of DNA–sufficiently short length and suitably fast analysis that the species and resistance genes can be identified in about an hour. Such a process has been proposed and demonstrated for the identification of resistance genes in bacteria associated with bloodstream infections (Sagar et al., 2018; Korshoj and Nagpal, 2019). This technique employs a block optical sequencing (BOS) method using surface-enhanced Raman spectroscopy (SERS) to obtain a spectrum of short DNA oligomers of length *k*, called *k*-mers (Sagar et al., 2018; Korshoj and Nagpal, 2019). Because the Raman spectrum of each A, T, G and C base is known, the overall ATGC content of a single *k*-mer can be calculated by mathematical analysis of the *k*-mer spectrum. Sequence information is lost, but the base content–called block optical content (BOC)–is preserved. For example, the 10 bp DNA segment ATATGGCCTT would become a BOC datum of A_2_T_4_G_2_C_2_. For very rapid analysis, this BOC technique can be multiplexed by creating an array of 1000s of pyramidal peaks on a silicon wafer whose entire peak field can be imaged by a sensitive CCD camera. Using band-pass filters at discrete spectral windows, optical intensity at specific wavelengths can be obtained simultaneously from all peaks. Finally the optical spectra are processed to obtain the ATGC content of the DNA on each pyramid peak. The size of the tips is such that 10 bases, but not any longer length, fit within the SERS electromagnetic field “hot-spot” (Sagar et al., 2018). It is estimated that using a 1,000 × 1,000 array of SERS pyramids on a silicon wafer, 1,000,000 reads of DNA 10-mers can be done in about 100 s, using high-throughput Raman spectroscopy using quantum dot optical filters (Bao and Bawendi, 2015) and digital processing of the resulting spectra.

Such a technique is ideally suited for genomic identification of bacteria, as a typical bacterial genome is about 5,000,000 bp, or 10 Mbase of single-stranded DNA (ssDNA). Clipping this genome into 10-mer lengths would provide enough ssDNA from a single bacterium to cover the SERS pyramids on a 1,000 × 1,000 array. Bloodstream infections contain very low counts of bacteria, often on the order of 10 colony forming units (CFU) per mL of blood. Thus a 10 mL sample of blood would provide 100-fold more DNA than needed to place a 10-mer on each pyramid. Bacteria (and their DNA) can be collected from blood in minutes (Pitt et al., 2016; Alizadeh et al., 2017; Pitt et al., 2019), and the SERS analysis can commence immediately, followed by computations for identification of species and antibiotic resistance.

Compared to species identification, analysis of resistance genes on plasmids is more challenging since those genes are usually contained within 500 to 1,000 bases while an average plasmid is roughly 200,000 bases in length. Thus, there is a much smaller signal to background ratio, making detection of resistance genes more difficult. Nevertheless, we show herein that our technique can still identify specific genes.

While such technology seems promising, a working device with many 1000s of pyramid tips is still in development, thus leaving some experimental questions unanswered for now. While we wait, however, many of the theoretical questions can be answered, the largest of which is whether the loss of sequence data (the BOC reads give only content, not sequence) will make it difficult to uniquely identify a bacterial species or state unequivocally whether a known resistance gene is present. Another theoretical question is whether random mutations in the bacterial genomes will compromise correct identification, or whether random noise from the experimental optical measurements will reduce accuracy.

The main goal of this study was to determine whether data produced in 10-mer blocks could be used in a diagnostic device, meaning that the data could correctly identify species and antibiotic resistance genes with a realistic number of pyramid tips (≤1,000,000 tips). To answer this question, this study addressed four main objectives: (1) to determine how many BOC reads are needed for species identification; (2) to determine how many BOC reads are needed for single gene detections (such as an antibiotic resistance gene of around 800 bp); (3) to analyze how accuracy is affected by noise from the detecting instrument and from random gene mutations; (4) to identify which learning algorithms are best at accurately identifying the species and genes.

The present study answers these and other questions to show that only 10^4^ BOC reads, even in the presence of mutations or experimental noise, are sufficient to identify bacterial species. The presence of resistance genes can be identified with an accuracy of 80% up to 95% using 10^5^ BOC reads. Both of these results are well below the proposed 10^6^ BOC reads, even when large error is present, showing that the algorithm could be used with high accuracy in a diagnostic device.

## Materials and Methods

**FIGURE 1:**
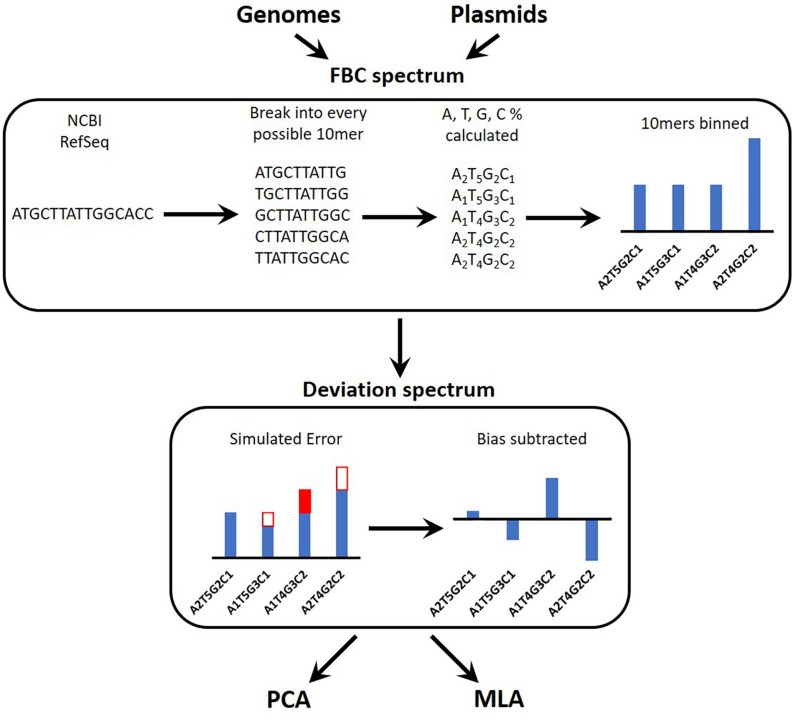
Overview of the identification algorithm. In actual experiments, bacterial DNA (genomic or plasmid) is digested into 10-base lengths. In simulated experiments and in teaching the learning algorithms, known DNA sequences are randomly broken into 10-base lengths. The block optical content (BOC) is measured for each 10-mer and put into bins corresponding to the fractional base composition (FBC) of each 10-mer, producing a distribution of FBC for the entire set of BOC measurements. In some cases random error is generated and added to (or subtracted from) the distribution. Finally the distribution of purely random ATCG 10-mers is subtracted from the FBC spectrum to produce a spectrum of deviation from randomness. This deviation spectrum is processed by PCA or the MLAs as described herein.

Figure 1 provides an overview of the process for simulating the experimental data reads, converting these into a spectrum, and testing the machine learning algorithms (MLAs). A brief outline follows: a sequence is obtained (see section “DNA Sequences”); the sequence is broken into every possible 10-mer for both strands of DNA (see section “Generating Sequence-Specific FBC Spectrum”); the 10-mers are binned according to the percent A, T, G, and C, resulting in the sequence-specific fractional base content (FBC) spectrum (see section “Generating Sequence-Specific FBC Spectrum”); experimental noise is simulated by introducing random errors into the sequence-specific FBC spectrum resulting in the simulated experimental FBC spectrum (see section “Simulating Gene Mutations and Experimental Errors”); the spectrum from a purely random sequence (bias) is subtracted from the simulated experimental FBC spectrum, producing a deviation spectrum (see section “Bias FBC Spectrum”); the FBC deviation spectra from many DNA samples are then analyzed by both principal component analysis (PCA) (see section “Principal Component Analysis”) and the MLAs (see section “Testing the MLAs”); the MLAs are trained and cross-validated on one set of FBC deviation spectra and then tested against another set of never-before-seen FBC deviation spectra (see section “Testing the MLAs”).

### DNA Sequences

While awaiting experimental data from the SERS instrument, simulated SERS BOC data were generated to determine the feasibility of the device in identifying bacterial species and antibiotic resistance genes. Reference genomes for 12 bacterial species, 728 plasmids containing 4 different types of carbapenem antibiotic-resistant genes, and 600 control plasmids not containing any carbapenem resistance genes were collected from the National Center of Biotechnology Information’s reference sequence (NCBI RefSeq; see Supplementary Material for NCBI reference IDs for each genome and plasmid) (O’Leary et al., 2016). The DNA sequences were separated into genomic and plasmid DNA (gDNA and pDNA, respectively) and studied separately using PCA and several MLAs.

Of the gDNA sequences, 10 of the 12 species are common organisms producing bloodstream infections (*Bacteroides fragilis, Campylobacter jejuni, Enterococcus hirae, Escherichia coli, Escherichia fergusonii, Klebsiella pneumoniae, Salmonella enterica, Staphylococcus aureus, Streptococcus pneumoniae*, and *Streptococcus pyogenes*) and were used in both training and testing (different genomic sequences were used for training and testing); and two additional species (*Klebsiella aerogenes* and *Mycobacterium tuberculosis*) were only used in testing (for taxonomy testing). Median GC% contents for the chosen species are included in Supplementary Table 4. One NCBI RefSeq genome was used for each species in the training set and one NCBI RefSeq genome was used for each species in the testing set. Only one was used because 1000 genomes are generated from each genome using the original genome FBC spectrum as the probability distribution for creating new genomes (see sections “Generating Sequence-Specific FBC Spectrum” and “Simulating Gene Mutations and Experimental Errors”). This means that no 2 simulated genomes are identical, even in the absence of error, and that the MLAs see the original genome and 999 variations for both the training and testing sets for each species, resulting in a similar analysis to one made with multiple NCBI RefSeq genomes per species.

For the pDNA, all sequenced plasmids from the NCBI RefSeq for the four carbapenem resistance plasmids [imipenemase 4 (IMP-4), Klebsiella pneumoniae carbapenemase 2 (KPC-2), New Delhi metallo-beta-lactamase 1 (NDM-1), and Verona integron-encoded metallobeta-lactamase 1 (VIM-1)] were used in either training or testing (see Table 1 for specific number used in training and testing). No variant plasmid data were used (a variant being KPC-4 or VIM-2), and all control plasmids were checked to make sure they did not contain the four carbapenem resistance plasmids or any variants. Two different tests were performed on the pDNA: the first test investigated whether the MLAs are able to identify the particular type of resistance (out of four types) or identify that none of these are present; the second test grouped the carbapenem resistance plasmids together and investigated whether the MLAs are able to identify the presence (or not) of any carbapenem resistance.

**TABLE 1:** Carbapenemase-gene-containing plasmids used in this study.

**Plasmid type**	**Number of plasmids used in training set**	**Number of plasmids in test set (not seen in training set)**
KPC-2	100	98
NDM-1	100	99
VIM-1	100	99
IMP-4	100	33
No-resistance plasmids	100 (or 400 for 2-group set)^a^	500 (or 200 for 2-group set)^a^

*^*a*^Plasmids were tested against individual resistant types and not resistant (five groups), or as a resistant vs. non-resistant grouping (two groups).*

### Generating Sequence-Specific FBC Spectrum

**FIGURE 2:**
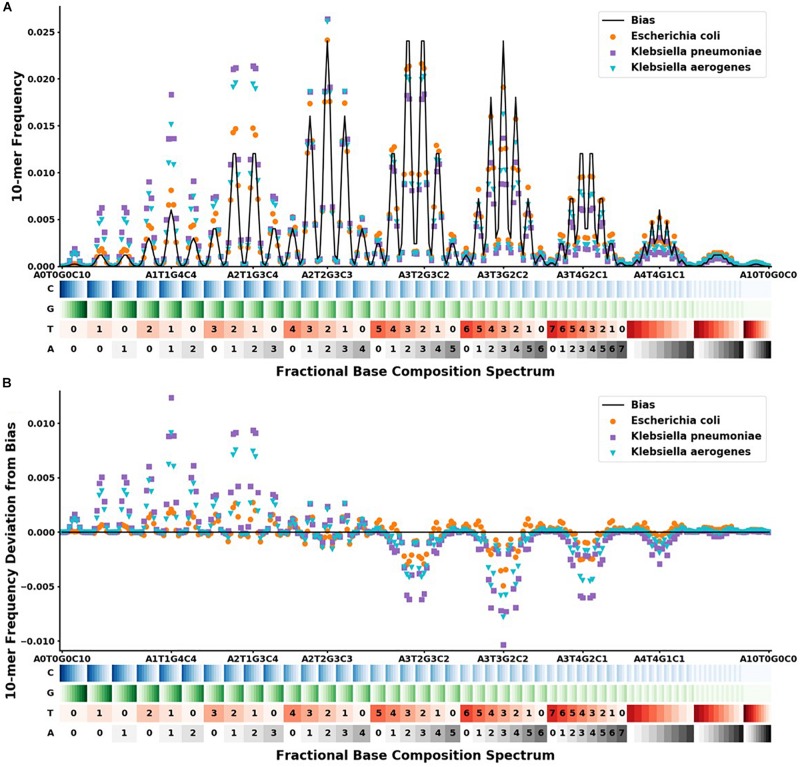
FBC spectra and deviation from bias FBC for *Klebsiella aerogenes* (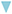), *Klebsiella pneumoniae* (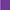), and *Escherichia coli* (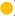). The x-axis shows the range of the 286 different FBC 10-mers starting with A_0_T_0_G_0_C_10_ and ending with A_10_T_0_G_0_C_0_. The A, T, G, and C’s are color-coded on a light to dark scale with the lightest shade representing 0 and the darkest shade representing 10. **(A)** Y-axis shows the frequency at which that FBC 10-mer appears in the genome sequence as a fraction of the total FBC 10-mer count; the black line indicates the normal random bias calculated from all possible FBC 10-mers. **(B)** Y-axis represents the deviation from normal randomness of the FBC 10-mer frequencies calculated by subtracting the bias from the frequencies counted in **(A)**. The graph shows that even closely related sequences have significantly different FBC spectra.

The physical optical instrument reads the BOC of each DNA *k*-mer bound to *r* number of SERS pyramids on a silicon chip in the instrument (Sagar et al., 2018). Since the size (i.e*., k* bases) of each read is known, these base fractions are converted into specific integer counts of nucleotides for each *k*-mer. BOC reads are written in the form A_*w*_T_*x*_G_*y*_C_*z*_ where 0 ≤ *w,x,y,z* ≤ *k*, and *w* + *x* + *y* + *z* = *k*. For traditional sequencing, there are 4*^*k*^* possible reads for a single *k*-mer; however, there are only (*k* + 3)!/(*k*!3!) BOC reads corresponding to the different ways of assigning the variables *w*, *x*, *y*, and *z*, given the previous constraints. The distribution of BOC reads, hereafter called the FBC spectrum, is defined as the probability distribution function of sampling any BOC read of a specific base composition (see Figure 2).

In order to create the FBC spectrum for each gDNA and pDNA sequences of interest, each sequence is decomposed into every possible 10-mer block using both complementary strands of DNA, since both strands will be present in a physical system and a 10-mer block from either strand has the same probability of adhering to the pyramid tip for the BOC reads. Therefore, in creating the FBC spectrum, the k-mers from both complementary strands of DNA are used. These blocks are then assigned to their corresponding bins to produce the FBC spectrum. Once all blocks are binned for the given sequence, each bin count is divided by the total 10-mer count of all 286 bins for that DNA sequence to get the sequence-specific probability distribution function, or FBC spectrum.

To simulate BOC reads on a multi-pyramid chip, the FBC spectrum for the selected plasmid or genome is randomly sampled 2.5 million times, using the sequence-specific probability distribution. This represents having more than one copy of the DNA sequence present in a physical experiment. From this 2.5-million-value array, the first *r* number of values are selected and distributed into bins to produce a simulated experimental FBC spectrum, where *r* represents the number of pyramid tips on the SERS-BOC device. This is done for both the training and testing sets.

### Simulating Gene Mutations and Experimental Errors

Due to mutations present in bacteria, any given bacteria species or plasmid will not have a perfectly identical FBC spectrum as that of the corresponding NCBI reference sequence. These natural gene mutations, which are on the order of 5 × 10^–4^ to 5 × 10^–9^ in bacteria (Denamur et al., 2002; Denamur and Matic, 2006), are expected to be overwhelmed by the experimental errors produced during experimental BOC reads and assignments. There are several sources for error in the optical sequencing reads. A few examples are under-digestion and/or over-digestion which results in non-uniform length *k*-mer sequences, *k*-mer sequences adhering to the pyramid tips such that not all bases can be read, portions of multiple *k*-mer sequences adhering to the same pyramid tip, and optical noise from the instrumentation (Sagar et al., 2018). While creating more realistic training and test samples, a single error rate parameter was introduced to modify the BOC read that accounts for both the expected bacterial mutations and the instrument errors, producing FBC spectra with various levels of random error.

We define an error rate *m* (where 0 ≤ *m* ≤ 1) to be the fraction of bases in the reference sequence that are expected to contain an error. Assuming that the errors are randomly distributed throughout the reference sequence, the number of errors in a randomly selected 10-mer is the same as the number of errors in 10 randomly selected bases. The probability of selecting a 10-mer without any errors is therefore determined by a binomial distribution in which the number of trials is the same as the number of pyramid tips, *r*, and the probability of being errorless is one minus the error rate, 1-*m*. To simulate errors, a value of either [0,1] is sampled from the binomial distribution B(*r,m*). If a 0 is chosen, then a value is chosen from the sequence-specific FBC spectrum (the 2.5-million-value array described above in Section “Generating Sequence-Specific FBC Spectrum”). If a 1 is chosen, then a random value from the bias spectrum (see section “Bias FBC Spectrum” for details) is chosen. This is repeated until a list of values is created that is *r* in length; next, the list is distributed into bins to produce the “noisy” FBC bins. The resulting FBC 10-mer bin counts are divided by *r* to obtain the “noisy” FBC spectrum for the given plasmid or genome.

### Bias FBC Spectrum

We discovered that a key to enhancing the differences in the FBC spectra of various DNA sequences is to subtract from each FBC spectrum the spectrum of totally random ATGC, leaving a spectrum of deviations from randomness. The resulting spectrum is called the “deviation spectrum” for a particular sequence.

Because the *k*-mer size is given, a purely random spectrum (called the bias spectrum) can be generated by including every possible *k*-mer once. Since there are 4*^*k*^* possible sequential *k*-mers, and given that any BOC read (A*_*w*_*T*_*x*_*G*_*y*_*C*_*z*_*) has *k*!/(*w*!*x*!*y*!*z*!) ways of permuting the base counts to create nucleotide-specific *k*-mers, the bias spectrum can be calculated as:

$$\mathrm{bias}\,\left(A_{w}\,T_{x}\,G_{y}\,C_{z}\right)=\frac{1}{4^{k}}\cdot \frac{k!}{w!\,x!\,y!\,z!}$$

This bias spectrum is subtracted from the FBC spectrum of the particular DNA sequence to yield a unique-DNA-sequence FBC deviation spectrum. This unique-DNA-sequence deviation spectrum is the deviation from pure randomness and should oscillate around zero.

For our given *k*-mer size of 10, there are 286 bins in the FBC spectrum. The FBC spectrum for a sequence can be visualized by plotting the frequency of a 10-mer in the bins of the spectrum, as shown in Figure 2. Figure 2A shows the corresponding FBC spectrum for *E. coli, K. pneumoniae, K. aerogenes*, and the bias spectrum. Figure 2B shows the resulting deviation spectra for *E. coli, K. pneumoniae*, and *K. aerogenes* produced by subtracting the bias spectrum. In Figure 2A, the commonness of the sequence is indicated by the height of the peak, with taller peaks being more common, such as A_3_T_2_G_3_C_2_ and A_3_T_3_G_2_C_2_. A few of these peaks are labeled for easy comparison with Figure 2B. As seen in Figure 2B, some FBC 10-mers appear more often than expected from a random sequence and some FBC 10-mers appear less than expected from a random sequence. While obvious that these bacteria do not have random sequences, it is useful and informative to observe that randomly breaking their DNA into all possible 10-mers does not produce a random FBC spectrum; in fact, unique features appear that suggest a species may be identified by its deviation spectrum.

To test the robustness of identification of species or genes by deviation spectra in the presence of real experimental noise and random mutations in the DNA sequence, random errors are introduced into the FBC spectra (see section “Simulating Gene Mutations and Experimental Errors” for details) and then the resulting noisy data are divided by *r* and the bias spectrum is subtracted to obtain a simulated FBC deviation spectrum (with noise) for the given plasmid or genome. For each gDNA and pDNA sequence (22 bacterial genomes and 1329 plasmids), 1000 simulated FBC deviation spectra were created from each specific sequence for both the training set and testing set.

### Principal Component Analysis

**FIGURE 3:**
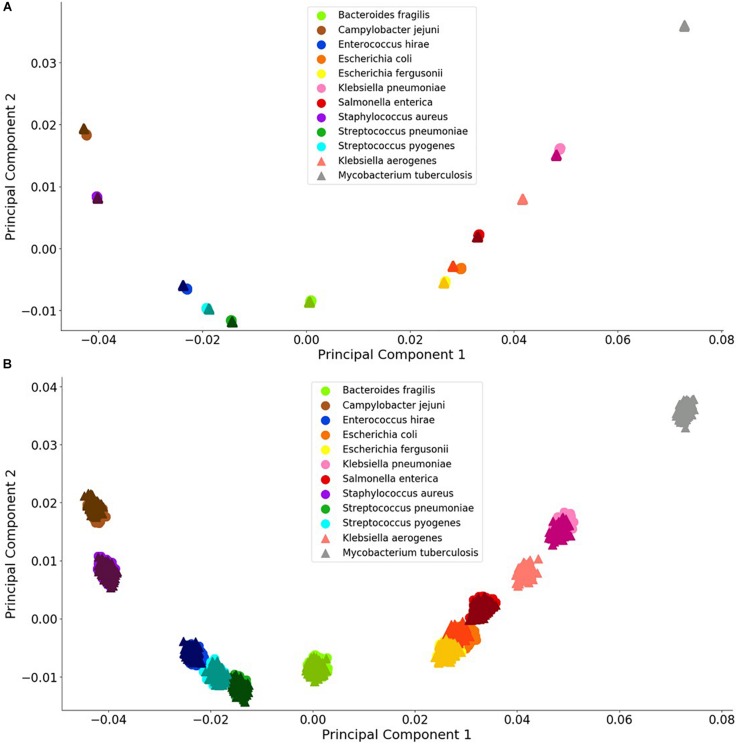
Visualization of bacterial species principal component analysis. Each of the 12 species tested is clustered individually. There are 1000 data points per species and each datum represents a single experiment with random error. The clusters indicate the distinctions in the FBC spectra. **(A)** 10^6^ BOC reads; **(B)** 10^4^ BOC reads. The error rate is 25% for both **(A,B)**. In both **(A,B)**, the darker triangles represent the never-before-seen genomes for the given species with the lighter circles representing the genomes trained on. The two additional triangles represent the extra species that the MLAs try to categorize according to the learning from the other nine species.

**FIGURE 4:**
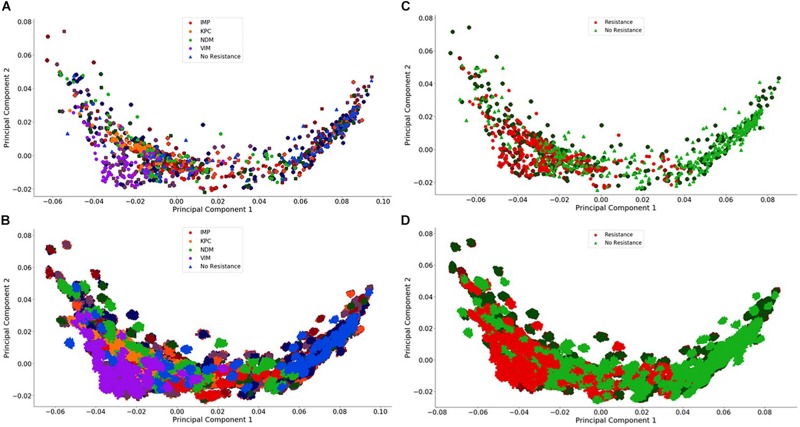
Visualization of the individual and group plasmid principal component analysis. The plasmid FBC spectra cannot be distinguished using only two principal components (500 FBC spectra per plasmid). **(A)** Individual 10^6^ BOC reads; **(B)** Individual 10^4^ BOC reads; **(C)** Group 10^6^ BOC reads; **(D)** Group 10^4^ BOC reads. The error rate is 0% for **(A–D)**. In **(A–D)**, the darker squares represent the never-before-seen plasmids for the given resistance type with the lighter circles representing the plasmids trained on. The non-resistant plasmids are represented by a darker diamond for the never-before-seen plasmids with a lighter triangle for training plasmids.

Principal component analysis is useful analysis is useful for reducing data dimensions data dimensions while retaining trends and patterns (Lever et al., 2017). This technique, which can reduce computational expense, is often used with biological data. For these reasons, PCA was used to investigate whether a simple data reduction analysis could easily identify the different species and antibiotic resistance genes using the noiseless and noisy FBC deviation spectra. As seen in Figures 3, 4, visualizing the first two principal components allows for some of the data to be easily classified into the correct groups, while other data are unclassifiable. Adding a third principal component may help cluster the data, but information is not easily retrieved from three dimensional plots, especially with 1000s of data points. From this initial result, MLAs were subsequently examined to determine if supervised learning methods could classify the data.

### Testing the MLAs

After creating the FBC deviation spectra from noiseless and noisy DNA BOC reads, the data sets were split into a training set and a never-before-seen test set (both sets of FBC spectra are created through the process detailed by Sections “Generating Sequence-Specific FBC Spectrum,” “Simulating Gene Mutations and Experimental Errors,” and “Bias FBC Spectrum”) and run through several MLAs to classify the bacterial species or the carbapenem resistance status of plasmids based on their FBC deviation spectra. The attributes used in the classification model were the values corresponding to the probability distribution from each FBC deviation spectrum. The training set was then randomly split into 900 and 100 deviation spectra for each DNA sequence for running 10-fold cross-validation. The MLAs were trained on the 900 deviation spectra from each DNA sequence and then tested against the remaining 100 deviation spectra from each DNA sequence of the training set and then tested against all of the never-before-seen sequences. For the gDNA, that meant that the MLAs were trained on 9,000 training deviation spectra (900 FBC spectra for each of the 10 training genome sequences) and validated against the remaining 1,000 training deviation spectra from the training set and then tested against all the 12,000 testing deviation spectra (1,000 FBC spectra for each of the 12 testing genome sequences). The MLAs were trained and tested similarly for the pDNA. For the gDNA, the labels used for classifying were: *B. fragilis, C. jejuni, E. hirae, E. coli, E. fergusonii, K. pneumoniae, S. enterica, S. aureus, S. pneumonia*, and *S. pyogenes*. The other two species, *K. aerogenes*, and *M. tuberculosis* are used to investigate how the MLAs group unknown species. For the pDNA, the labels used for the individual classification tests were: KPC, NDM, VIM, IMP and No Resistance; and the labels used for the group classification tests were: Resistance and No Resistance.

For both the gDNA and pDNA, the FBC deviation spectra were created with the following parameters: *k* = 10; *r* = [10^2^; 10^3^; 10^4^; 10^5^; 10^6^]; *m* = [0, 0.01, 0.05, 0.1, 0.25, 0.33, 0.5, 0.75, 0.9, 1]; and *s* = 1000; where *k* is the size of the *k*-mer, *r* is the number of pyramid tips for generating the sample FBC spectra, *m* is the fractional error rate, and *s* is the number of FBC deviation spectra created per DNA sequence (genome or plasmid). All 50 combinations of *r* and *m* were tested.

From the machine learning python package, Sci-kit learn (version 0.20.3) (Pedregosa et al., 2011), 11 different MLAs were tested from the following categories: linear MLAs, decision tree learning algorithms, Naïve Bayes learning algorithms, discriminant analyses, and a neural network. For this initial study, default parameters were used for all of these algorithms. Each of these classification models were chosen for their ability to fit data with positive and negative values and to fit data to the model using out-of-core fitting, except for the discriminant analyses (see Supplementary Material for further details). Presented in the results is the best algorithm from each category; the results of the other algorithms are in the Supplementary Material.

### Model Performance Testing and Statistical Analysis

The robustness of each classification model was studied by measuring the predictive accuracy as a function of the parameters of the simulated BOC data. For each optical sequencing read number (*r*) and each error rate (*m*), the performance of the model was quantified by the predictive accuracy and a confusion matrix, which keeps track of the true positives, true negatives, false positives, and false negatives for the sample (Powers, 2011). The accuracies and confusion matrix presented for each MLA are the average of 10 trials (*n* = 10) for the given *r* and *m*. Three different cutoff accuracies (95% for species, 90% for group plasmid, and 75% for individual plasmid) for the never-before-seen sequences were chosen as criteria for assessing the effects of the different error rates and sequencing read numbers.

### Simulation

To generate large amounts of simulated experimental data on which to test the different MLAs, the data were produced using code written in Python 3.7 as described above. Reference genomes were downloaded directly from the NCBI RefSeq database. All code used for running the simulation is available at: https://github.com/rlwphd/DNAFingerprints. While these simulations were tested only on plasmids having a carbapenem antibiotic resistance gene, the simulation will work for any set of antibiotic resistance genes in which the full sequence of the gene-containing plasmid is known.

## Results

### Principal Component Analysis

Figures 3, 4 visually display the first two principal components for the deviation spectra of the species and resistance genes. For species, the PCA data are visually distinct, even when significant noise is added to the BOC data. For resistance gene detection, the PCA could not produce a clear distinction. Details are discussed below.

For both bacterial species and antibiotic resistance gene identification, it is noted that the PCA produced in all cases contains an arch effect, which indicates that the principal components are not completely independent of each other and are thus not completely orthogonal to each other (Morton et al., 2017). Since the PCA assumes independence and orthogonality between the principal components, it is not completely reliable as a means of identification without first adjusting for the arch effect. However, PCA was used here to reveal the extent of differences that might be learned by the MLAs.

#### Bacterial Species

For the bacterial species, we found that the PCA revealed significant differences between the various species, even with a 90% error rate for 10^6^ reads. As a control, using a 100% error rate produced no differences (data not shown) because the PCA yields the same value for all species, regardless of the number of reads. We selected *E. coli* and *E. fergusonii*, two genetically very similar bacteria, as a stringent test for sensitivity and discrimination. Because of the similarity of *E. coli* and *E. fergusonii*, the graphical plot can only reveal the difference between these two species for error rates of 0–33% for 10^5^ reads and 0–90% for 10^6^ reads (see difference between Figures 3A,B). Figure 3 contains an example of a non-overlapping (significantly different) PCA (A) and an overlapping (some difference to no difference) PCA (B). Figure 3 shows the PCA of the FBC deviation spectra data for the bacteria species for 25% error at 10^6^ reads (A) and at 10^4^ reads (B) (see Supplementary Material for additional PCA figures). This figure shows that with enough BOC reads even in the presence of error, there exists significant differences between the species when using only the first two principal components. This indicates that the FBCs of each of these 12 species are distinct enough that the MLAs should be able to easily classify each species, potentially perfectly, even in the presence of noise, even differentiating very similar species such as *E. coli* and *E. fergusonii*.

#### Antibiotic Resistance Genes

For the plasmids, the PCA revealed that there is a greater spread across the plasmid sequences with no clear differences using only two principal components for any number of reads, even with no error. This is not surprising because the antibiotic resistant gene content could be affecting less than 1% of the FBC deviation spectrum (800 bases on a 200,000-base plasmid) for some of the samples. This indicates that the MLAs will need to detect the small resistance gene signal from a dominating background. In addition to a low signal competing with a strong background, there is a wide range of different DNA signatures in plasmids, increasing the complexity of the task. Figure 4 highlights this varying range of DNA signatures; both control plasmids and plasmids containing resistance genes span the entire space of the PCA plot. Neither the individual resistance categorization (Figures 4A,B) nor the group resistance categorization (Figures 4C,D) provided any insights into clustering or separation. Figure 4 shows the PCA of the FBC spectra data for the plasmids for 0% error at 10^6^ reads (A&C) and at 10^4^ reads (B&D) (see Supplementary Material for additional PCA figures).

### Classification Using MLAs

The outputs from 1000s of simulated SERS-BOC experiments were produced to examine a wide range of combinations of error rates and the number of reads from pyramid tips. The simulation creates hypothetical BOC reads from different bacterial genomes or plasmids which are then converted to FBC deviation spectra and handed to the different MLAs for categorizing. Simulating the experiments allows us to show feasibility and test the robustness of the algorithm in the presence of noise and genetic variation, and to examine the device as a diagnostic tool for bacteria classification and resistance profiling.

The results detailed below showed that the best algorithms from each category are: Passive-Aggressive Classifier (PA, linear machine learning algorithm); Extra Trees Classifier (ET, decision tree algorithm); Gaussian Naïve Bayes (GNB, Naïve Bayes algorithm); Linear Discriminant Analysis (LDA, discriminant analysis algorithm); and the neural network (NN, whose default is 100 layers and the number of nodes determined by the number of input features). The results for each of these MLAs are presented below and the results for the other MLAs can be found in Supplementary Tables 1–3.

#### Bacterial Species

The MLA analysis showed that we were able to accurately classify greater than 96% of the simulated unseen bacterial genome data sets (1000 samples per species) using the ET, LDA and NN MLAs employing as low as 10^4^ BOC reads and up to 50% error. The PA MLA was the worst algorithm and never got better than 78% accuracy (see Supplementary Material for detailed table). As for the best algorithms, even at 90% error, the LDA and NN could accurately classify greater than 98% of the bacterial species at 10^5^ BOC reads. This surprising result at very high noise levels is postulated to occur because random error added to the BOC generates random noise in the FBC spectrum, but the deviation spectrum has the bias spectrum subtracted out which removes random noise. Thus, the MLA models working on the FBC deviation spectra operate on data with a good signal-to-noise level, even up through 90% random error in the original BOC data.

**FIGURE 5:**
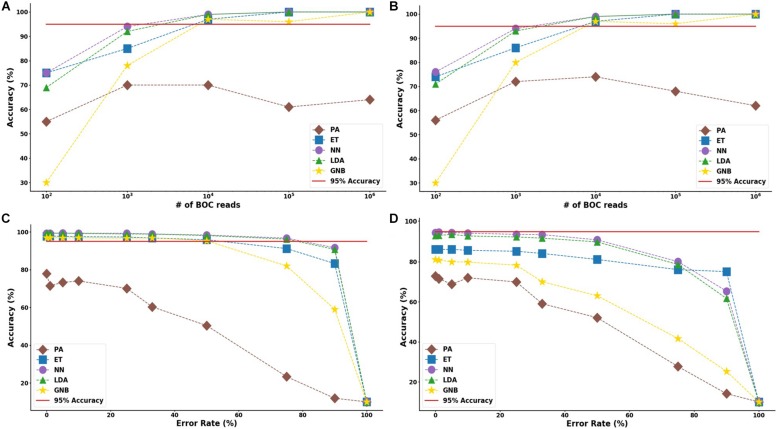
Species model accuracy for different number of BOC reads and error rates. The accuracy of the five MLAs is shown with **(A)** 10% error, and **(B)** 25% error in identifying the never-before-seen bacterial species sets at different number of BOC reads. The accuracy of the five MLAs is shown with **(C)** 10^4^ BOC reads, and **(D)** 10^3^ BOC reads in identifying the never-before-seen bacterial species sets at different error rates. The red line represents 95% accuracy threshold.

The results suggest that a SERS-BOC device only needs a 100 × 100 pyramid array (∼10^4^ reads per experiment) to accurately identify bacterial species. Figure 5 shows the MLA accuracy for an error rate of 10% (A) and 25% (B) for all of the BOC reads. This figure shows that greater than 97% accuracy is maintained at 10^4^ BOC reads for both 10 and 25% error. Figure 5 also shows how the various MLAs hold up to error for 10^4^ BOC reads (C) and 10^3^ BOC reads (D).

**FIGURE 6:**
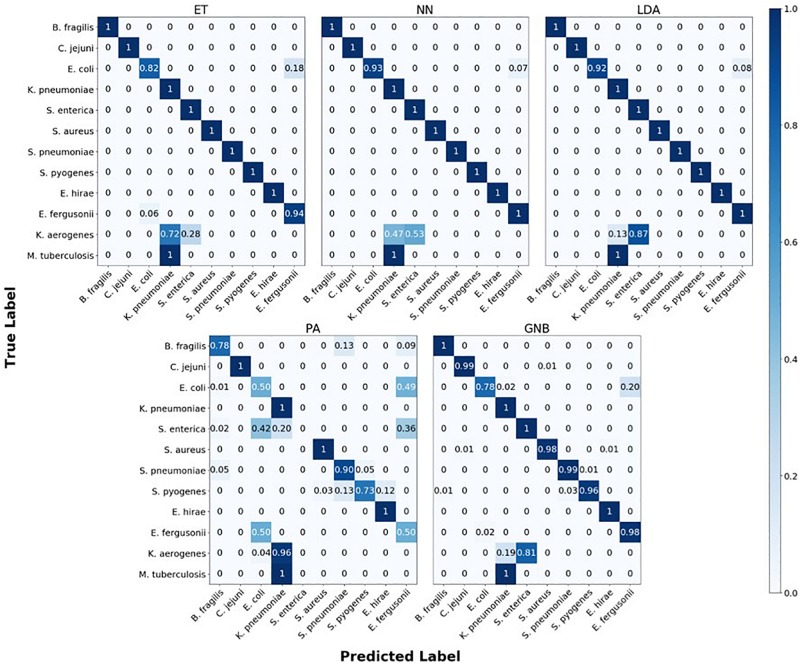
Confusion matrix for the five MLAs for species identification. *r* = 10^4^, *m* = 0.10. The x-axis represents the label predicted by the algorithm and the y-axis represents the true label.

The confusion matrix for the MLAs shows how the models mislabel the species they were trained on, and how they label the two species which are not defined in the model. Figure 6 shows the species confusion matrix for the five MLAs for 10^4^ BOC reads and 10% error. While most of the species in this study are not closely related (see Supplementary Material for details), the best three MLAs only had problems distinguishing between *E. coli* and *E. fergusonii*, which are genetically very similar (same genus). The ET had a sensitivity rate of 82% for *E. coli* and a specificity rate of 94% for *E. fergusonii* when comparing the two genomes. The LDA had a sensitivity rate of 92% for *E. coli* and a specificity rate of 100% for *E. fergusonii* when comparing the two genomes; meaning that the LDA could correctly identify *E. fergusonii* but not *E. coli*. The NN had a sensitivity rate of 93% for *E. coli* and a specificity rate of 100% for *E. fergusonii* when comparing the two genomes. All other genomes trained on had 100% identification for the ET, LDA, and NN MLAs. As for the classification of the two extra species (*K. aerogenes* and *M. tuberculosis*), all three MLAs identify *M. tuberculosis* as *K. pneumoniae* (unrelated) 100% of the time. *K. aerogenes* was identified as both *K. pneumoniae* (same genus) and *S. enterica* (same family) by all three MLAs. Other details are found in Figure 6.

#### Antibiotic Resistance Genes

**FIGURE 7:**
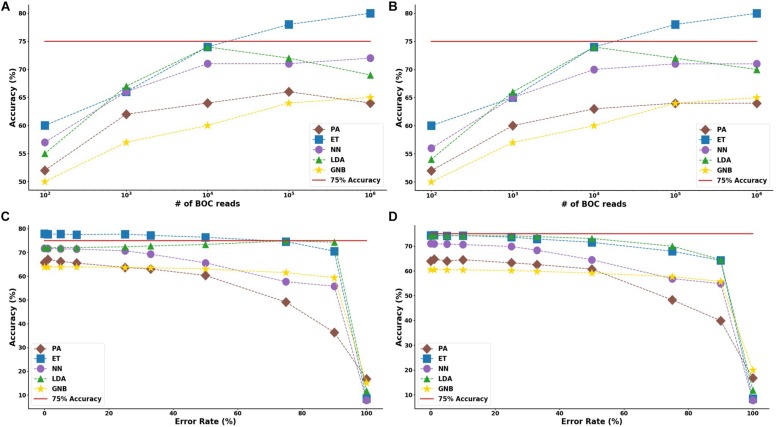
Individual antibiotic-resistance model accuracy for different number of BOC reads and error rates. The accuracy of the five MLAs is shown with **(A)** 10% error, and **(B)** 25% error in identifying the never-before-seen plasmid sets for individual identification. The accuracy of the five MLAs is shown with **(C**) 10^5^ BOC reads, and **(D)** 10^4^ BOC reads in identifying the never-before-seen plasmid sets for individual identification. The red line represents a 75% accuracy threshold.

In the study of the individual resistance categorization (distinguishing four carbapenem resistance genes), the MLAs had difficulty achieving better than 75% accuracy. Only with 10^5^ BOC reads (or more) did the ET algorithm attain better than 75% accuracy, and even this algorithm only achieved 80% accuracy at best. The LDA and ET algorithms had similar accuracy at 10^4^ reads (see Figure 7D) but the LDA did not perform as well at 10^5^ reads (Figure 7C). The GNB and PA MLAs were the worst but were still able to achieve 64% accuracy (see Figure 7 and Supplementary Material for a detailed table). Figure 7 shows the MLA accuracy for an error rate of 10% (A) and 25% (B) for all of the BOC reads for the individual scenarios. Figures 7A,B show that greater than 75% accuracy is maintained at 10^5^ BOC reads for both 10 and 25% error. Figure 7 also shows how the MLAs hold up to error for 10^5^ BOC reads (C) and 10^4^ BOC reads (D).

**FIGURE 8:**
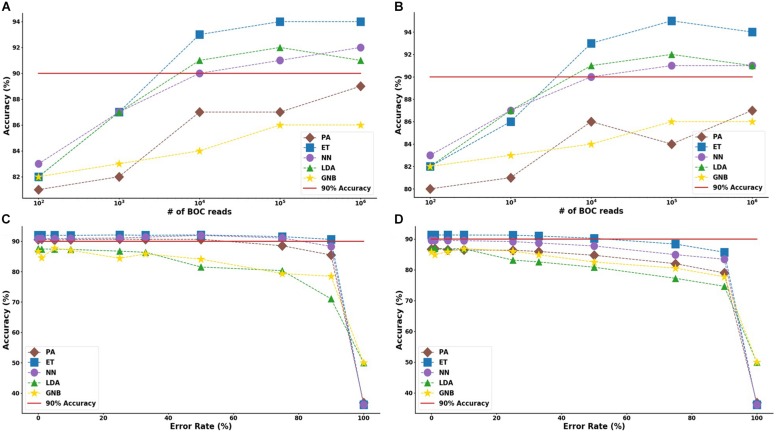
Group antibiotic-resistance model accuracy with different number of BOC reads and error rates. The accuracy of the five MLAs is shown with **(A)** 10% error, and **(B)** 25% error in identifying the never-before-seen plasmid sets for group identification. The accuracy of the five MLAs is shown with **(C)** 10^5^ BOC reads, and **(D)** 10^4^ BOC reads in identifying the never-before-seen plasmid sets for group identification. The red line represents a 90% accuracy threshold.

For the group resistance categorization, the MLA analysis was able to accurately classify 90% of the simulated unseen data set using the ET, LDA and NN MLAs down to 10^4^ BOC reads and up to 50% error. The GNB MLA performed the worst of those examined but was still able to achieve 86% accuracy (see Figure 8 and Supplementary Material for detailed table). Figure 8 shows the MLA accuracy for an error rate of 10% (A) and 25% (B) for all of the BOC reads for the group scenario. Figure 8 shows that greater than 90% accuracy is maintained at 10^4^ BOC reads for both 10 and 25% error. Figure 8 also shows how the MLAs hold up to error for 10^5^ BOC reads (C) and 10^4^ BOC reads (D).

The ET MLA model for the individual resistance categorization maintains 74% accuracy for up to 25% error for 10^4^ BOC reads and maintains 80% accuracy for up to 25% error for 10^6^ BOC reads. The individual results suggest that a SERS-BOC device would need to be at least a 100 × 100 pyramid array (10^4^ reads per experiment), but a 1,000 × 1,000 pyramid array (10^6^ reads per experiment) would be optimal for the best results to accurately identify antibiotic resistance genes on plasmids. For the group resistance categorization, the three best MLA models maintain good signal-to-noise levels up through 50% error. The group results suggest that a SERS-BOC device with a 100 × 100 pyramid array would be sufficient to accurately identify whether any carbapenem resistance gene was present in the presence of 50% experimental noise.

**FIGURE 9:**
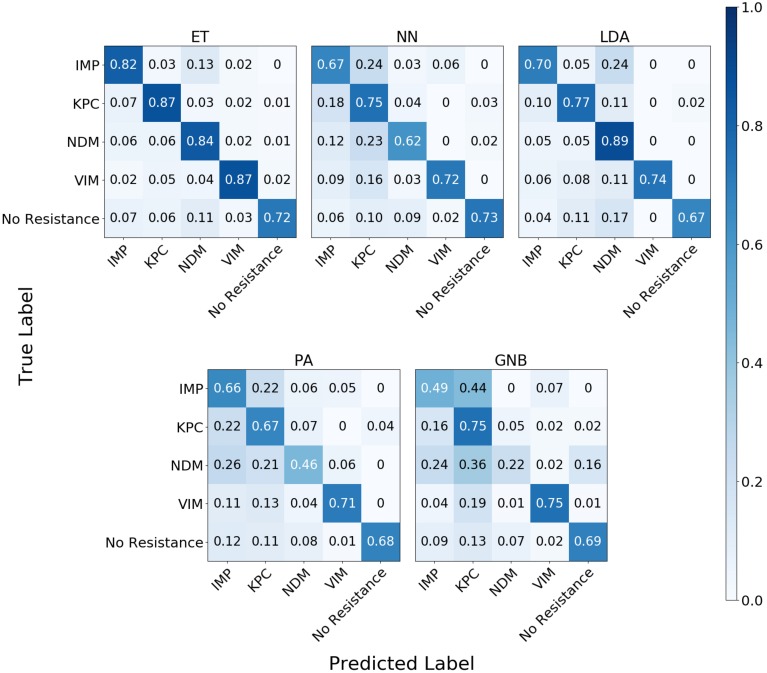
Confusion matrix for the five MLAs for individual identification of the plasmid sets. *r* = 10^5^, *m* = 0.10 The x-axis represents the label predicted by the algorithm and the y-axis represents the true label.

**FIGURE 10:**
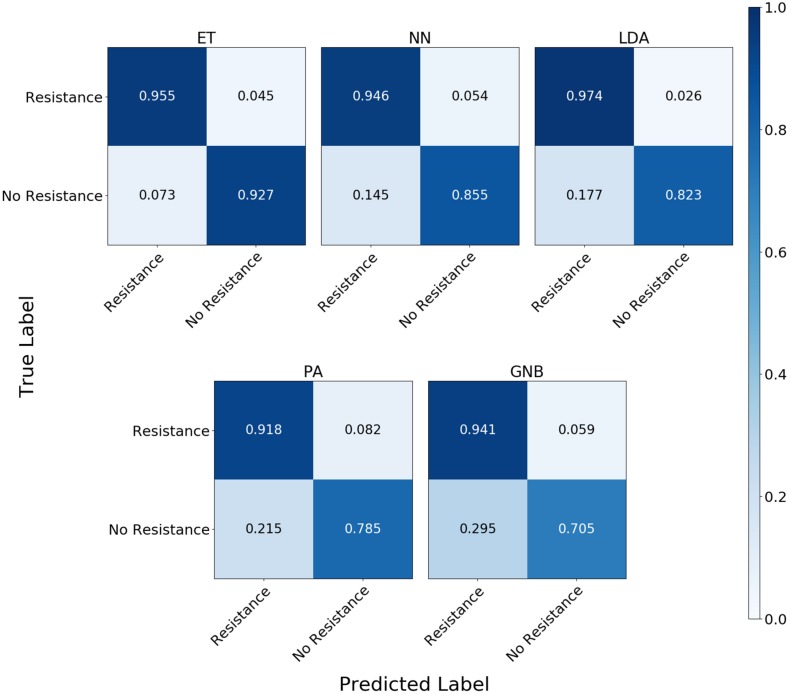
Confusion matrix for the five MLAs for group identification of the plasmid sets. *r* = 10^5^, *m* = 0.10 The x-axis represents the label predicted by the algorithm and the y-axis represents the true label.

The confusion matrix for the MLAs shows how the models mislabel the plasmids, which allows us to calculate the false-negative rate for the antibiotic-resistant plasmids as a group, as well as the sensitivity of each individual plasmid. The false-negative rate metric is clinically important as it is incorrectly labeling resistant plasmids as not being resistant, which could result in the wrong (ineffective) antibiotics being given to the patient, possibly leading to death. The sensitivity metric shows which type of resistance gene is harder to identify. Figure 9 shows the confusion matrix for the five MLAs for 10^5^ BOC reads and 10% error for the plasmid sets for individual identification. Figure 10 shows the confusion matrix for the five MLAs for 10^5^ BOC reads and 10% error for the plasmid sets for group identification.

The sensitivity rate at 10^5^ BOC reads and 10% error is the values on the diagonal on the confusion matrices shown in Figures 9, 10. The clinically important false negative rates at 10^5^ BOC reads with 10% error from the individual carbapenem resistance identification are 0.50% for LDA, 1.00% for ET, 1.25% for NN, 2.50% for PA, and 4.75% for GNB. The false-negative rates at 10^5^ BOC reads with 10% error from the grouped carbapenem resistance identification are 2.6% for LDA, 4.5% for ET, 5.4% for NN, 5.9% for GNB, and 8.2% for PA. Thus, the LDA MLA appears best at avoiding false-negative errors when identifying resistance genes on plasmids, while the ET most accurately identifies the resistance genes.

## Discussion

This research is the first of its kind and validates the SERS-BOC instrument (currently in development) as an excellent predictor of genetic signatures, even in the presence of genetic mutations and experimental noise. The use of FBC deviation spectra was able to achieve greater than 99% accuracy in classifying bacterial species using several types of MLAs.

We were able to detect individual antibiotic resistance genes on plasmids from their FBC deviation spectra with 80% accuracy and to detect the presence of a carbapenem resistance gene with 94% accuracy using MLAs. The implications of our research suggest that the use of MLA classifiers on fractional base composition data generated from a SERS-BOC type instrument (Sagar et al., 2018) has tremendous potential in accurately identifying both bacterial species and antibiotic resistance genes. With respect to bloodstream infection diagnosis, the creation of FBC models has the potential to help determine the bacterial species and the antibiotic-resistance profile associated with a bloodstream infection in a cost-efficient and time-efficient way, thus improving the outcomes for patients. Of most import, the false negative evaluations for carbapenem resistance genes were less than 3% using the LDA algorithm with 10^5^ BOC reads.

We note with surprise the lack of spread within a species data cluster in PCA analysis even when introducing a 25% error rate. This indicates that the inclusion of error does not introduce enough variance to cause these species to have overlap in the principal component space until the number of BOC reads get low (10^2^ and 10^3^ reads). Because we are sampling at least a million different *k*-mers to generate the FBC spectrum, the distribution of any single training sample does not deviate significantly from the FBC of the non-mutated genome. The high number of reads available on a 1-million-pyramid SERS array would enable this high predictive ability, and may be more than necessary. Even with experimental error causing deviation from the reference FBC, the FBCs of different species are distinct enough that the simulated experimental noise had no negative effect on clustering in principal component space. The random error in BOC data from genomic mutation and experimental noise in the FBC spectra are subtracted out since we know what the random spectrum looks like. Thus, random errors reduce the signal levels but have little effect on noise levels.

The MLA analysis shows how robust the species model is at handling experimental noise in the FBC, allowing for substantial error rates while maintaining its predictive power. Again, one reason why the model can still accurately classify species even with high error rates is that the experimental noise mimics the random bias distribution. Since we subtract out this random bias before running the machine learning classifier, we are essentially “subtracting out” the effects of the mutation or noise, and what is left is a smaller size of the accurate FBC, which the MLA can still use to accurately classify the species of bacteria. For example, 10^6^ BOC reads with 90% random errors still leaves 10^5^ good reads once the randomness is subtracted.

This study challenged the algorithms with much more random error in the data than expected in actual experiments. The largest source of error is anticipated to be wrong assignment of the BOC values due to spectroscopic noise. Currently the accuracy of calling correct BOC is about 90% (Sagar et al., 2018), but accuracy is improving with time and experience and optical quality. Thus 25% error is probably a gross overestimate of actual experimental error, which we anticipate will be on the order of 10% or less. Random point mutations in bacteria are far less than 0.1% and have no bearing on the accuracy of species identification.

Plasmids come in all different sizes ranging from 2,000 to 200,000 bp long (and longer), and the carbapenem-resistant genes are about 800 bp. This means that the FBC that comes from the resistant genes has varying weight upon the FBC spectrum of the entire plasmid, which would explain why there is so much spread from one resistant plasmid to another in the PCA analysis. The ability for the MLAs to learn from that 800-bp region amongst all of the other data and noise shows great promise for this type of antibiotic resistance gene identification. Overall, the never-before-seen results show that the good performance of the models is not a result of overfitting; rather, the models are actually learning how to recognize the characteristic signatures in an FBC deviation spectrum when a specific target gene is included. Of course the application of the algorithm for antibiotic resistance identification requires knowledge of an antibiotic resistance gene sequence, so this technique would not identify novel antibiotic resistance evolved by mutations.

In a real-world application, these models would need to be tuned to perform optimally, but the low sensitivity of the accuracy to error rates ranging from 0 to 90% proves the robustness of the models in dealing with noise. As previously stated, we postulate that this robustness stems from the subtraction of randomness from within the data set. A high number of BOC reads with high random error is similar to a model having less SERS-BOC readings, which our results show is still effective down to the 10^4^ BOC reads. Additional sources of error in a real-world setting could include DNA contamination (human or bacterial), lack of good separation between plasmids and genomes (which would make the resistant genes on plasmids less distinguishable), and untested or new bacterial species causing the blood infections. We anticipate the continued development of the SERS-BOC instrument that will provide real BOC data from which FBC data can be created to further test our assumptions and postulates. Further application of this method could go beyond analyzing bacterial bloodstream infections into other clinical scenarios that need fast and reliable analysis of only a few key genes of known sequence.

There are known limitations to this study. The first and foremost is that all of the data had to be simulated because a large enough device to produce experimental data are still being developed. Another limitation is that only a single group of antibiotic resistance genes (carbapenem resistant genes) was tested. Therefore, the results cannot be broadcasted to other groups of resistance genes or to identifying other genes. Also, this technique can only identify antibiotic resistance through known DNA sequences. Another limitation is the limited number of bacterial species used. Because of the focus on testing the technology for clinical use in bloodstream infections, a select group of bacterial species was used for this initial study. Future studies will test the ability of this technique to identify between all species. This study also did not tune the MLAs or perform a cost-benefit analysis to identify the factors influencing the accuracy of the MLAs. However, this study provides the initial groundwork for further exploration in using a SERS-BOC device to identify species and genes based on 10-mer DNA sequences.

## Data Availability Statement

All datasets generated for this study are included in the article/Supplementary Material.

## Author Contributions

RW contributed to the concept, ran the simulations, and contributed to the writing. TJ and CW contributed to the concept and ran initial simulations. MC advised the simulations and contributed to the writing. PN contributed to the concept and contributed to the writing. WP contributed to the concept, advised the simulations, and contributed to the writing.

## Conflict of Interest

The authors declare that the research was conducted in the absence of any commercial or financial relationships that could be construed as a potential conflict of interest.
