# Supplementary material for: Analysis of Identification Method for Bacterial Species and Antibiotic Resistance Genes Using Optical Data From DNA Oligomers
Source: Front Microbiol. 2020 Feb 20;11:257. doi: 10.3389/fmicb.2020.00257 (PMC7044133; doi:10.3389/fmicb.2020.00257)
Supplement: Supplementary file 1 [file Data_Sheet_1.PDF]

Supplementary Material

1 Machine Learning Algorithms

The following machine learning algorithms were tested: 3 linear machine learning algorithms were tested with default parameters Stochastic Gradient Descent Classifier (SGD), Passive Aggressive Classifier (PA), and Perceptron (Per); 3 decision tree machine learning algorithms were tested with default parameters Random Forest (RF), Gradient Boosting Classifier (GB), and Extra Trees Classifier (ET); 2 naïve bayes algorithms were tested with default parameters Bernoulli Naïve Bayes (BNB) and Gaussian Naïve Bayes (GNB); 2 discriminant analyses were tested with default parameters Linear Discriminant Analysis (LDA) and Quadratic Discriminant Analysis (QDA); and 1 neural network with default parameters.

The 2 discriminant analyses were tested due to the results of the PCA figures in an initial analysis of the genomes. Theses algorithms can only train on 250,000 data samples and cannot use out-of-core learning. Since there was only 10 bacterial species, the LDA and QDA were trained on the full training set. Due to there being 1329 plasmids, the LDA and QDA could not be trained on the full training set. Therefore, the data was randomly selected, such that there was an even number of each type of antibiotic resistance (or resistance grouping) in the training set. However, the LDA and QDA were tested against the full never-before-seen set.

2 Data for all error rate and BOC reads combinations for all MLAs

Supplementary Table 1. MLA accuracy for the different error rates and number of BOC reads for bacterial species for the never before seen genomes. (The 5 used MLAs are bolded.)

# of BOC reads	Error	**PA**	Per	SGD	**ET**	GB	RF	BNB	**GNB**	**LDA**	QDA	**NN**
100	100%	0.10	0.10	0.10	0.10	0.10	0.10	0.10	0.10	0.10	0.10	0.10
1000	100%	0.10	0.10	0.10	0.10	0.10	0.10	0.10	0.10	0.10	0.10	0.10
10000	100%	0.10	0.10	0.10	0.10	0.10	0.10	0.10	0.10	0.10	0.10	0.10
100000	100%	0.10	0.10	0.10	0.10	0.10	0.10	0.10	0.10	0.10	0.10	0.10
1000000	100%	0.10	0.10	0.10	0.10	0.10	0.10	0.10	0.10	0.10	0.10	0.10
100	90%	0.13	0.13	0.20	0.67	0.29	0.64	0.32	0.24	0.36	0.47	0.39
1000	90%	0.14	0.17	0.17	0.75	0.45	0.55	0.45	0.25	0.62	0.61	0.65
10000	90%	0.12	0.19	0.17	0.83	0.72	0.68	0.75	0.59	0.91	0.77	0.92
100000	90%	0.14	0.17	0.16	0.94	0.93	0.88	0.91	0.77	0.98	0.74	0.99
1000000	90%	0.12	0.33	0.18	0.97	0.89	1.00	0.94	0.61	1.00	0.68	1.00
100	75%	0.30	0.22	0.22	0.73	0.38	0.60	0.43	0.25	0.48	0.40	0.52
1000	75%	0.28	0.27	0.22	0.76	0.56	0.54	0.66	0.42	0.79	0.68	0.80
10000	75%	0.23	0.36	0.20	0.91	0.84	0.79	0.87	0.82	0.96	0.89	0.97
100000	75%	0.24	0.36	0.21	0.99	0.97	0.97	0.95	0.84	0.99	0.90	1.00
1000000	75%	0.23	0.34	0.21	1.00	0.94	0.99	1.00	0.91	1.00	0.91	1.00
100	50%	0.44	0.32	0.38	0.75	0.45	0.55	0.55	0.26	0.61	0.58	0.63
1000	50%	0.52	0.43	0.40	0.81	0.67	0.64	0.80	0.63	0.90	0.75	0.91

10000	50%	0.50	0.58	0.39	0.96	0.92	0.88	0.94	0.95	0.98	0.85	0.98
100000	50%	0.52	0.49	0.44	0.99	0.98	0.97	0.96	0.92	1.00	0.97	1.00
1000000	50%	0.51	0.44	0.43	1.00	0.91	1.00	0.97	1.00	1.00	0.96	1.00
100	33%	0.53	0.43	0.42	0.75	0.47	0.54	0.62	0.29	0.68	0.61	0.72
1000	33%	0.59	0.56	0.49	0.84	0.74	0.67	0.84	0.70	0.92	0.79	0.94
10000	33%	0.60	0.60	0.49	0.97	0.92	0.91	0.94	0.97	0.99	0.88	0.99
100000	33%	0.59	0.59	0.50	0.99	0.98	0.98	0.96	0.93	1.00	0.97	1.00
1000000	33%	0.58	0.50	0.53	1.00	0.91	1.00	0.95	1.00	1.00	1.00	1.00
100	25%	0.55	0.43	0.47	0.75	0.49	0.54	0.63	0.30	0.69	0.62	0.75
1000	25%	0.70	0.52	0.49	0.85	0.75	0.69	0.85	0.78	0.92	0.76	0.94
10000	25%	0.70	0.68	0.55	0.97	0.94	0.92	0.95	0.97	0.99	0.91	0.99
100000	25%	0.61	0.61	0.50	1.00	0.98	0.99	0.97	0.96	1.00	1.00	1.00
1000000	25%	0.64	0.43	0.55	1.00	0.91	1.00	0.90	1.00	1.00	1.00	1.00
100	10%	0.56	0.44	0.48	0.74	0.51	0.53	0.66	0.30	0.71	0.62	0.76
1000	10%	0.72	0.57	0.53	0.86	0.77	0.71	0.85	0.80	0.93	0.82	0.94
10000	10%	0.74	0.62	0.57	0.97	0.93	0.93	0.96	0.97	0.99	0.91	0.99
100000	10%	0.68	0.62	0.55	1.00	0.99	0.99	0.98	0.96	1.00	0.99	1.00
1000000	10%	0.62	0.47	0.59	1.00	0.91	0.99	0.89	1.00	1.00	0.98	1.00
100	5%	0.57	0.48	0.49	0.74	0.50	0.53	0.65	0.30	0.71	0.63	0.77
1000	5%	0.69	0.58	0.53	0.86	0.78	0.71	0.86	0.80	0.94	0.78	0.95
10000	5%	0.73	0.57	0.57	0.98	0.94	0.93	0.96	0.97	0.99	0.88	0.99
100000	5%	0.69	0.61	0.49	1.00	0.99	0.99	0.98	0.97	1.00	1.00	1.00
1000000	5%	0.69	0.49	0.50	1.00	0.91	0.99	0.87	1.00	1.00	1.00	1.00
100	1%	0.57	0.48	0.46	0.74	0.50	0.53	0.66	0.31	0.72	0.62	0.77
1000	1%	0.72	0.59	0.53	0.86	0.78	0.70	0.86	0.81	0.93	0.78	0.95
10000	1%	0.72	0.60	0.55	0.98	0.94	0.93	0.96	0.97	0.99	0.91	0.99
100000	1%	0.69	0.65	0.55	1.00	0.99	0.99	0.98	0.97	1.00	0.99	1.00
1000000	1%	0.74	0.49	0.51	1.00	0.90	0.99	0.88	1.00	1.00	1.00	1.00
100	0%	0.54	0.48	0.49	0.74	0.51	0.54	0.66	0.31	0.71	0.63	0.76
1000	0%	0.73	0.54	0.53	0.86	0.79	0.71	0.86	0.81	0.93	0.81	0.95
10000	0%	0.78	0.64	0.57	0.98	0.94	0.93	0.96	0.97	0.99	0.87	0.99
100000	0%	0.71	0.67	0.54	1.00	0.99	0.99	0.98	0.98	1.00	0.98	1.00
1000000	0%	0.70	0.48	0.51	1.00	0.90	1.00	0.87	1.00	1.00	1.00	1.00

Supplementary Table 2. MLA accuracy for the different error rates and number of BOC reads for the antibiotic resistances with individual resistance categorization for the never before seen plasmids. (The 5 used MLAs are bolded.)

# of BOC reads	Error	**PA**	Per	SGD	**ET**	GB	RF	BNB	**GNB**	**LDA**	QDA	**NN**
100	100%	0.20	0.20	0.20	0.20	0.20	0.20	0.20	0.20	0.20	0.20	0.20
1000	100%	0.20	0.20	0.20	0.20	0.20	0.20	0.20	0.20	0.20	0.20	0.20
10000	100%	0.20	0.20	0.20	0.20	0.20	0.20	0.20	0.20	0.20	0.20	0.20
100000	100%	0.20	0.20	0.20	0.20	0.20	0.20	0.20	0.20	0.20	0.20	0.20
1000000	100%	0.20	0.20	0.20	0.20	0.20	0.20	0.20	0.20	0.20	0.20	0.20
100	90%	0.36	0.23	0.29	0.57	0.37	0.54	0.48	0.35	0.46	0.20	0.49
1000	90%	0.41	0.23	0.38	0.60	0.51	0.54	0.53	0.45	0.53	0.29	0.52
10000	90%	0.40	0.39	0.39	0.64	0.56	0.60	0.56	0.56	0.65	0.49	0.55
100000	90%	0.36	0.31	0.39	0.71	0.64	0.67	0.59	0.59	0.74	0.63	0.56
1000000	90%	0.37	0.29	0.40	0.77	0.70	0.72	0.63	0.63	0.75	0.79	0.56
100	75%	0.48	0.26	0.51	0.60	0.47	0.53	0.52	0.42	0.49	0.26	0.51
1000	75%	0.50	0.39	0.54	0.61	0.53	0.56	0.56	0.52	0.58	0.38	0.55
10000	75%	0.48	0.43	0.55	0.68	0.58	0.65	0.59	0.58	0.70	0.50	0.57
100000	75%	0.49	0.42	0.55	0.75	0.68	0.70	0.62	0.62	0.75	0.71	0.58
1000000	75%	0.50	0.42	0.56	0.78	0.73	0.74	0.63	0.64	0.72	0.85	0.58
100	50%	0.52	0.43	0.57	0.60	0.50	0.54	0.54	0.49	0.52	0.36	0.53
1000	50%	0.55	0.50	0.62	0.64	0.55	0.60	0.58	0.55	0.63	0.38	0.59
10000	50%	0.61	0.52	0.62	0.72	0.63	0.68	0.61	0.59	0.73	0.58	0.65
100000	50%	0.60	0.53	0.63	0.76	0.70	0.72	0.64	0.63	0.73	0.76	0.66
1000000	50%	0.59	0.53	0.63	0.79	0.75	0.75	0.64	0.65	0.70	0.87	0.66
100	33%	0.52	0.35	0.58	0.60	0.51	0.54	0.55	0.50	0.54	0.36	0.55
1000	33%	0.58	0.57	0.62	0.65	0.56	0.61	0.60	0.57	0.65	0.48	0.64
10000	33%	0.63	0.59	0.63	0.73	0.65	0.69	0.62	0.60	0.74	0.62	0.68
100000	33%	0.63	0.60	0.64	0.77	0.71	0.73	0.64	0.64	0.73	0.77	0.69
1000000	33%	0.62	0.60	0.64	0.79	0.75	0.75	0.64	0.65	0.70	0.87	0.69
100	25%	0.52	0.43	0.58	0.60	0.52	0.55	0.56	0.50	0.54	0.37	0.56
1000	25%	0.60	0.57	0.63	0.65	0.56	0.61	0.60	0.57	0.66	0.48	0.65
10000	25%	0.63	0.62	0.64	0.74	0.65	0.69	0.63	0.60	0.74	0.63	0.70
100000	25%	0.64	0.64	0.65	0.78	0.72	0.72	0.64	0.64	0.72	0.78	0.71
1000000	25%	0.64	0.64	0.65	0.80	0.75	0.74	0.64	0.65	0.70	0.87	0.71
100	10%	0.52	0.40	0.58	0.60	0.52	0.55	0.56	0.50	0.55	0.36	0.57
1000	10%	0.62	0.59	0.63	0.66	0.57	0.62	0.60	0.57	0.67	0.49	0.66
10000	10%	0.64	0.66	0.64	0.74	0.66	0.69	0.63	0.60	0.74	0.64	0.71
100000	10%	0.66	0.67	0.65	0.78	0.72	0.73	0.64	0.64	0.72	0.79	0.71
1000000	10%	0.64	0.67	0.65	0.80	0.75	0.74	0.64	0.65	0.69	0.87	0.72
100	5%	0.54	0.40	0.58	0.60	0.52	0.55	0.56	0.50	0.55	0.36	0.57
1000	5%	0.62	0.59	0.63	0.66	0.57	0.62	0.60	0.57	0.67	0.50	0.67
10000	5%	0.65	0.66	0.65	0.74	0.66	0.69	0.63	0.60	0.74	0.65	0.71

100000	5%	0.66	0.67	0.65	0.78	0.72	0.73	0.63	0.64	0.72	0.79	0.72
1000000	5%	0.65	0.67	0.65	0.80	0.76	0.74	0.64	0.65	0.69	0.87	0.72
100	1%	0.55	0.41	0.58	0.60	0.52	0.55	0.56	0.50	0.55	0.36	0.57
1000	1%	0.62	0.59	0.63	0.66	0.57	0.62	0.61	0.57	0.67	0.50	0.67
10000	1%	0.65	0.66	0.65	0.74	0.66	0.69	0.63	0.60	0.74	0.65	0.71
100000	1%	0.67	0.68	0.65	0.78	0.72	0.74	0.63	0.64	0.72	0.79	0.72
1000000	1%	0.66	0.68	0.65	0.80	0.76	0.73	0.64	0.65	0.69	0.87	0.72
100	0%	0.54	0.43	0.58	0.60	0.52	0.55	0.56	0.50	0.55	0.37	0.57
1000	0%	0.62	0.59	0.63	0.66	0.57	0.62	0.61	0.57	0.67	0.50	0.67
10000	0%	0.64	0.66	0.65	0.74	0.66	0.69	0.63	0.60	0.74	0.65	0.71
100000	0%	0.66	0.68	0.65	0.78	0.72	0.74	0.63	0.64	0.72	0.79	0.72
1000000	0%	0.66	0.68	0.65	0.80	0.77	0.73	0.64	0.65	0.69	0.87	0.72

Supplementary Table 3. MLA accuracy for the different error rates and number of BOC reads for the antibiotic resistances with group resistance categorization for the never before seen plasmids. (The 5 used MLAs are bolded.)

# of BOC reads	Error	**PA**	Per	SGD	**ET**	GB	RF	BNB	**GNB**	**LDA**	QDA	**NN**
100	100%	0.50	0.50	0.50	0.50	0.50	0.50	0.50	0.50	0.50	0.50	0.50
1000	100%	0.50	0.50	0.50	0.50	0.50	0.50	0.50	0.50	0.50	0.50	0.50
10000	100%	0.50	0.50	0.50	0.50	0.50	0.50	0.50	0.50	0.50	0.50	0.50
100000	100%	0.50	0.50	0.50	0.50	0.50	0.50	0.50	0.50	0.50	0.50	0.50
1000000	100%	0.50	0.50	0.50	0.50	0.50	0.50	0.50	0.50	0.50	0.50	0.50
100	90%	0.76	0.69	0.37	0.78	0.71	0.76	0.74	0.76	0.76	0.69	0.76
1000	90%	0.79	0.70	0.37	0.82	0.80	0.76	0.81	0.82	0.81	0.78	0.82
10000	90%	0.78	0.75	0.37	0.86	0.84	0.79	0.83	0.83	0.86	0.83	0.84
100000	90%	0.78	0.71	0.37	0.92	0.88	0.85	0.81	0.84	0.91	0.88	0.85
1000000	90%	0.77	0.71	0.37	0.94	0.90	0.90	0.80	0.85	0.91	0.91	0.86
100	75%	0.76	0.65	0.52	0.80	0.75	0.76	0.79	0.80	0.80	0.73	0.81
1000	75%	0.81	0.75	0.47	0.83	0.83	0.77	0.82	0.83	0.83	0.83	0.83
10000	75%	0.81	0.77	0.46	0.89	0.86	0.82	0.82	0.84	0.88	0.85	0.87
100000	75%	0.79	0.80	0.46	0.94	0.89	0.89	0.78	0.85	0.92	0.91	0.89
1000000	75%	0.80	0.81	0.46	0.94	0.90	0.91	0.78	0.86	0.90	0.91	0.89
100	50%	0.80	0.74	0.82	0.82	0.79	0.76	0.82	0.82	0.81	0.79	0.82
1000	50%	0.82	0.77	0.83	0.85	0.84	0.79	0.82	0.83	0.85	0.84	0.85
10000	50%	0.83	0.81	0.83	0.91	0.88	0.85	0.79	0.84	0.90	0.88	0.89
100000	50%	0.84	0.82	0.83	0.94	0.90	0.91	0.78	0.85	0.92	0.92	0.90
1000000	50%	0.84	0.81	0.83	0.94	0.92	0.90	0.78	0.86	0.90	0.88	0.90
100	33%	0.79	0.76	0.82	0.82	0.81	0.76	0.82	0.82	0.81	0.82	0.82
1000	33%	0.81	0.78	0.83	0.86	0.85	0.80	0.82	0.83	0.86	0.84	0.86
10000	33%	0.85	0.83	0.83	0.92	0.88	0.86	0.78	0.84	0.91	0.89	0.90
100000	33%	0.86	0.86	0.83	0.94	0.90	0.91	0.78	0.85	0.92	0.91	0.91

1000000	33%	0.86	0.86	0.83	0.94	0.92	0.90	0.78	0.86	0.91	0.88	0.91
100	25%	0.80	0.76	0.83	0.82	0.81	0.76	0.82	0.82	0.82	0.82	0.83
1000	25%	0.81	0.77	0.83	0.86	0.85	0.80	0.81	0.83	0.87	0.84	0.87
10000	25%	0.86	0.83	0.83	0.93	0.89	0.86	0.78	0.84	0.91	0.89	0.90
100000	25%	0.84	0.87	0.83	0.95	0.90	0.91	0.78	0.86	0.92	0.91	0.91
1000000	25%	0.87	0.86	0.83	0.94	0.91	0.89	0.78	0.86	0.91	0.87	0.91
100	10%	0.81	0.77	0.83	0.82	0.82	0.76	0.82	0.82	0.82	0.82	0.83
1000	10%	0.82	0.79	0.83	0.87	0.85	0.80	0.81	0.83	0.87	0.84	0.87
10000	10%	0.87	0.87	0.83	0.93	0.89	0.86	0.78	0.84	0.91	0.89	0.90
100000	10%	0.87	0.87	0.83	0.94	0.91	0.91	0.78	0.86	0.92	0.91	0.91
1000000	10%	0.89	0.88	0.83	0.94	0.92	0.89	0.77	0.86	0.91	0.87	0.92
100	5%	0.80	0.76	0.83	0.82	0.82	0.76	0.82	0.82	0.82	0.82	0.83
1000	5%	0.82	0.80	0.83	0.87	0.85	0.80	0.81	0.83	0.87	0.84	0.87
10000	5%	0.86	0.87	0.83	0.93	0.89	0.87	0.78	0.84	0.91	0.90	0.90
100000	5%	0.86	0.87	0.83	0.95	0.91	0.91	0.78	0.86	0.92	0.91	0.91
1000000	5%	0.88	0.88	0.83	0.94	0.92	0.90	0.77	0.86	0.91	0.87	0.92
100	1%	0.79	0.76	0.83	0.82	0.82	0.76	0.82	0.82	0.82	0.82	0.83
1000	1%	0.82	0.82	0.83	0.87	0.85	0.80	0.81	0.83	0.87	0.84	0.87
10000	1%	0.85	0.87	0.83	0.93	0.89	0.87	0.78	0.84	0.91	0.90	0.90
100000	1%	0.85	0.87	0.83	0.95	0.91	0.91	0.78	0.86	0.92	0.91	0.91
1000000	1%	0.87	0.88	0.83	0.94	0.91	0.90	0.77	0.86	0.91	0.87	0.92
100	0%	0.80	0.76	0.83	0.82	0.82	0.76	0.82	0.82	0.82	0.82	0.83
1000	0%	0.81	0.82	0.83	0.87	0.85	0.80	0.81	0.83	0.87	0.84	0.87
10000	0%	0.86	0.87	0.83	0.93	0.89	0.87	0.78	0.84	0.91	0.90	0.90
100000	0%	0.87	0.88	0.83	0.94	0.91	0.91	0.78	0.86	0.92	0.91	0.91
1000000	0%	0.87	0.88	0.83	0.94	0.91	0.91	0.77	0.86	0.91	0.87	0.92

3 100 BOC reads

In creating the random bias, there are 4^k possible sequential *k*-mers which for *k*=10 gives 1,048,576 *k*-mers. When we get down to 100 and 1000 BOC reads, we are greatly under-sampling the bias FBC. Therefore, in order to ensure that the bias remains true to the bias sequence, we checked the bias by sampling 100 different random 100 BOC reads (see Supplemental Figure 1) and compared them against the bias FBC (see Supplemental Figure 2). Then we created an FBC from the 100 random samples by summing the bins for the 100 samples and compared that FBC to the bias FBC (see Supplemental Figure 3). From Supplemental Figure 1, we see that no one sample covers the entire FBC space, but collectively they begin to sample the entire space. From Supplemental Figure 2, we see that the individual samples do not match the bias FBC. It is higher in some regions and lower in others. But from Supplemental Figure 3, the summed FBC from the 100 samples matches the bias FBC.

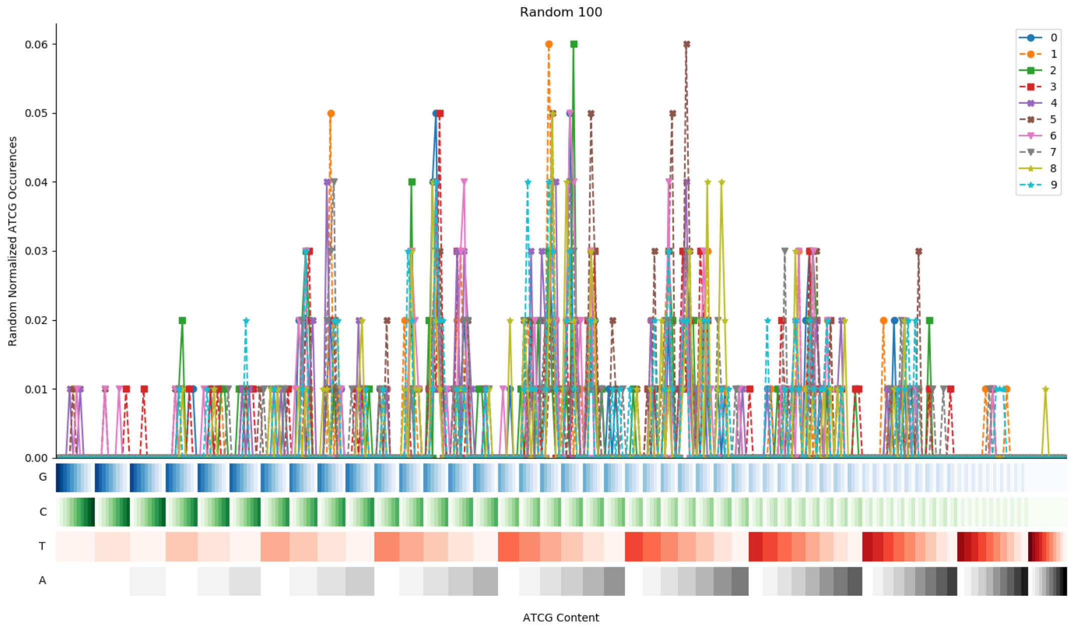

Supplemental Figure 1. Ten of the 100 randomly sampled 100 BOC reads from the bias FBC.

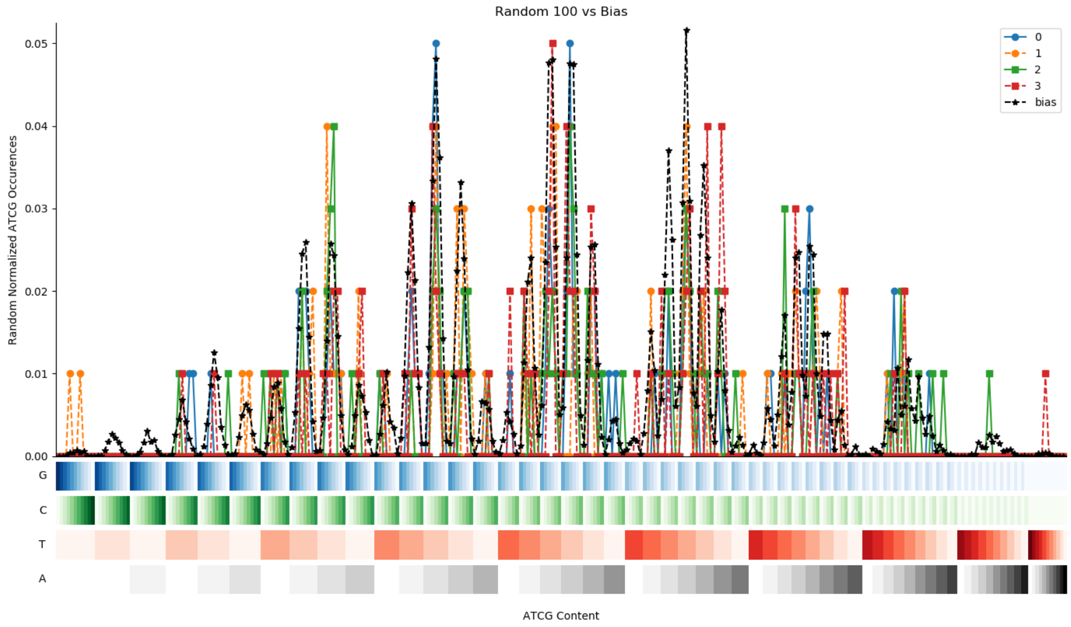

Supplemental Figure 2. Four of the 100 randomly sampled 100 BOC reads from the bias FBC compared to the bias FBC.

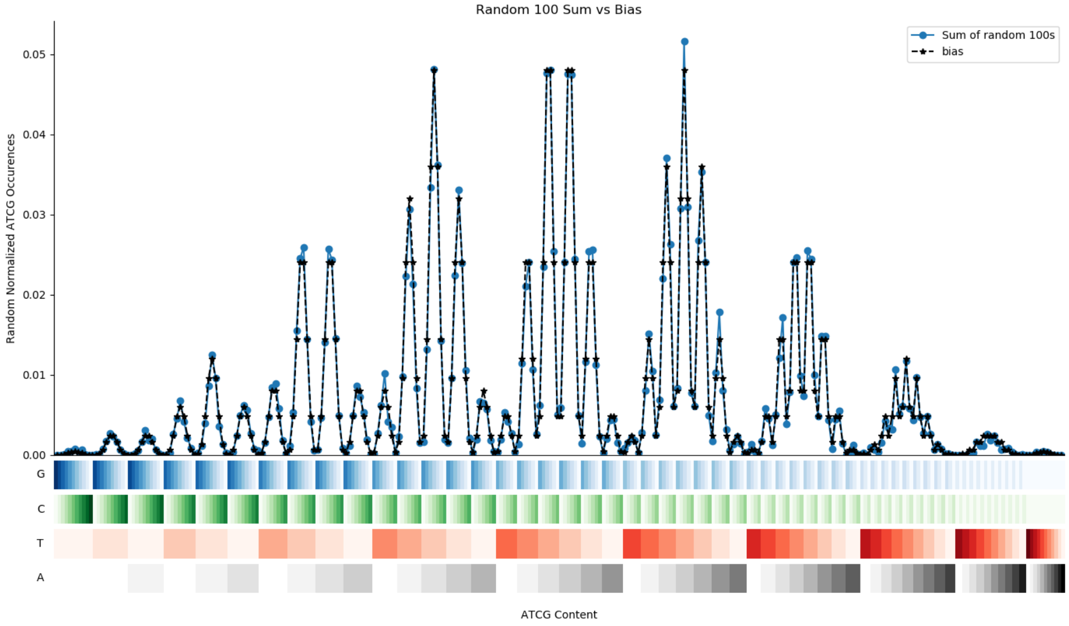

Supplemental Figure 3. The FBC from summing the 100 randomly sampled 100 BOC reads from the bias FBC compared to the bias FBC.

4 NCBI Reference IDs

Supplementary Table 4. NCBI Reference IDs and GC% for the genomes.

Bacteria	Training	Testing	% GC
Bacteroides fragilis	NC_006347.1	CP011073.1	43.4%
Campylobacter jejuni	NC_002163.1	NC_003912.7	30.4%
Escherichia coli	U00096.3	AE005174.2	50.6%
Escherichia fergusonii	NC_011740.1	NZ_CP040805.1	49.9%
Salmonella enterica	NC_003197.2	CP006631.1	52.1%
Klebsiella pneumoniae	NC_012731.1	FO834906.1	57.2%
Staphylococcus aureus	NC_007622.1	BX571856.1	32.7%
Enterococcus hirae	LR134297.1	NC_018081.1	36.8%
Streptococcus pyogenes	NC_002737.1	AE014074.1	38.4%
Streptococcus pneumoniae	NC_003028.3	CP000919.1	39.6%
Klebsiella aerogenes	-	NC_015663.1	55.0%
Mycobacterium tuberculosis	-	AP018035.1	65.6%

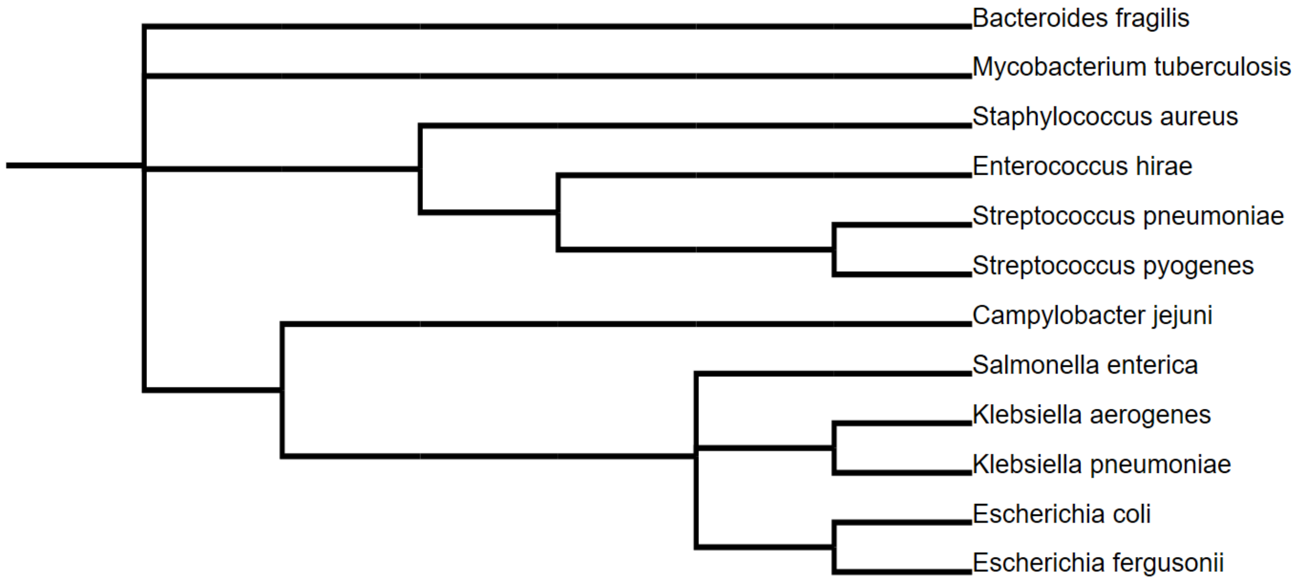

Supplementary Figure 4. Taxonomy of the bacterial species pulled from the NCBI database to show how the species are related.

Supplementary Table 5. NCBI Reference IDs for the plasmids.

IMP-4	KPC-2	NDM-1	VIM-1	Non-resistant		
NC_019368.1	NC_011382.1	NC_015872.1	NC_014368.1	NC_018988.1	NC_010262.1	NC_007682.3
NC_022652.1	NC_011383.1	NC_016974.1	NC_019375.1	NC_013379.2	NC_010260.1	NC_008259.1
NG_049203.1	NC_014312.1	NC_016980.1	NC_019906.1	NC_013393.1	NC_010330.1	NC_010064.1
NZ_CP016763.1	NC_016846.1	NC_018994.1	NC_021576.1	NC_013383.1	NC_007190.1	NC_010261.1
NZ_CP021328.1	NC_019152.1	NC_019045.2	NC_021622.1	NC_013313.1	NC_004429.1	NC_010282.1
NZ_CP022533.1	NC_019161.1	NC_019063.1	NC_022242.1	NC_018997.1	NC_007203.1	NC_010377.1
NZ_CP024192.1	NC_019161.1	NC_019069.1	NC_024987.1	NC_019005.1	NC_002773.1	NC_010859.1
NZ_CP024522.1	NC_019384.1	NC_019089.1	NC_025186.1	NC_013391.1	NC_010378.1	NC_014508.2
NZ_CP024529.1	NC_020132.1	NC_019153.1	NC_032101.1	NC_018965.1	NC_010409.1	NC_015056.1
NZ_CP024557.1	NC_020893.1	NC_019158.1	NG_050336.1	NC_013341.1	NC_010481.1	NC_006903.1
NZ_CP025964.2	NC_021238.1	NC_019162.1	NZ_ATHX01000046.1	NC_013305.1	NC_010426.1	NC_007594.1
NZ_CP028486.1	NC_021660.2	NC_019163.1	NZ_CM010663.1	NC_013307.1	NC_010427.1	NC_007773.1
NZ_CP028952.1	NC_021664.2	NC_019268.1	NZ_CP023419.1	NC_013345.1	NC_009781.1	NC_002776.1
NZ_CP031121.1	NC_022078.1	NC_019281.1	NZ_CP023442.1	NC_013346.1	NC_002147.1	NC_003969.1
NZ_JQNT01000104.1	NC_022346.1	NC_019360.1	NZ_CP023926.1	NC_013308.1	NC_010021.1	NC_011227.1
NZ_JRFQ01000062.1	NC_022885.1	NC_019889.1	NZ_CP025467.1	NC_013312.1	NC_010077.1	NC_012220.1
NZ_JTEB01000058.1	NC_023331.1	NC_019985.2	NZ_CP026661.1	NC_013292.1	NC_010099.1	NC_001763.1
NZ_JTGO01000050.1	NC_023903.1	NC_020552.1	NZ_CP029718.1	NC_013293.1	NC_003099.1	NC_004334.1
NZ_JUBD01000040.1	NC_023904.1	NC_020811.1	NZ_CP030081.1	NC_013299.1	NC_008821.1	NC_004653.1
NZ_JUBE01000054.1	NC_023905.1	NC_020818.1	NZ_CP031570.1	NC_013301.1	NC_001399.1	NC_011409.1
NZ_JUGC01000052.1	NC_023906.1	NC_021180.1	NZ_CP031573.1	NC_019008.1	NC_005570.1	NC_007206.1

NZ_KQ089505.1	NC_023907.1	NC_021198.1	NZ_CP031576.1	NC_013340.1	NC_006868.1	NC_008486.1
NZ_LAIY01000158.1	NC_024967.1	NC_021501.1	NZ_CP031584.1	NC_019009.1	NC_006904.1	NC_014233.1
NZ_LFXT01000042.1	NC_025019.1	NC_022589.1	NZ_CP031610.1	NC_013342.1	NC_001272.2	NC_015068.1
NZ_LFXU01000060.1	NC_025166.1	NC_022609.1	NZ_CP032168.1	NC_013344.1	NC_006399.1	NC_015849.1
NZ_LRJV01000033.1	NC_025167.1	NC_023322.1	NZ_CP032176.1	NC_018982.1	NC_010616.1	NC_015860.1
NZ_LRJW01000124.1	NC_025183.1	NC_023908.1	NZ_CP032178.1	NC_018983.1	NC_010684.1	NC_015861.1
NZ_LRJX01000101.1	NC_025187.1	NC_023911.1	NZ_CP034084.1	NC_018984.1	NC_010685.1	NC_015863.1
NZ_NGAG01000014.1	NC_032098.1	NC_023914.1	NZ_FWYI01000067.1	NC_018986.1	NC_010686.1	NC_015864.1
NZ_NGAH01000052.1	NC_032103.1	NC_024954.1	NZ_JYGA02000003.1	NC_018987.1	NC_010687.1	NC_015862.1
NZ_NGAI01000045.1	NG_049253.1	NC_024959.1	NZ_LIDY01000091.1	NC_018998.1	NC_010726.1	NC_015872.1
NZ_NGAJ01000013.1	NZ_ANGI02000157.1	NC_024999.1	NZ_LLWB01000129.1	NC_013392.2	NC_007635.1	NC_015900.1
NZ_NGAK01000052.1	NZ_AOGQ01000049.1	NC_025000.1	NZ_LNGO01000036.1	NC_013300.1	NC_004959.1	NC_015901.1
NZ_NGAL01000047.1	NZ_AP018674.1	NC_025106.1	NZ_LNHV01000004.1	NC_013395.1	NC_001774.1	NC_015902.1
NZ_NIII01000041.1	NZ_APVO01000006.1	NC_025116.1	NZ_LPOA01000052.1	NC_019033.1	NC_010851.1	NC_015912.1
NZ_NIIJ01000054.1	NZ_APVP01000035.1	NC_025130.1	NZ_LPOE01000075.1	NC_018985.1	NC_010857.1	NC_001446.1
NZ_NIIK01000054.1	NZ_APVQ01000147.1	NC_025184.1	NZ_LPOF01000059.1	NC_019040.1	NC_010861.1	NC_009137.1
NZ_NIIR01000049.1	NZ_APVR01000051.1	NG_049326.1	NZ_LPOH01000002.1	NC_019041.1	NC_010862.1	NC_011266.1
NZ_NPGF01000059.1	NZ_APVS01000045.1	NZ_AGFH01000030.1	NZ_LPOI01000051.1	NC_011917.1	NC_010870.1	NC_011522.1
NZ_NWEC01000032.1	NZ_APVT01000059.1	NZ_AOCV01000085.1	NZ_LPOQ01000121.1	NC_019037.1	NC_010885.1	NC_010098.1
NZ_PCFT01000003.1	NZ_APVV01000098.1	NZ_AP014649.1	NZ_LPPA01000096.1	NC_019039.1	NC_010886.1	NC_004971.1
NZ_PTXQ01000049.1	NZ_APVY01000038.1	NZ_AP018750.1	NZ_LPPL01000133.1	NC_019044.1	NC_010898.1	NC_004163.1
NZ_PWAQ01000079.1	NZ_APVZ01000026.1	NZ_AP018753.1	NZ_LPPN01000057.1	NC_019043.1	NC_010913.1	NC_005247.1
NZ_PWAR01000086.1	NZ_APWA01000117.1	NZ_AVAN01000031.1	NZ_LPPS01000099.1	NC_019053.1	NC_010919.1	NC_006979.1
NZ_PWAS01000080.1	NZ_APWB01000186.1	NZ_BDHK01000043.1	NZ_LPPT01000038.1	NC_019054.1	NC_010929.1	NC_013121.1
NZ_RDRD01000120.1	NZ_APWD01000124.1	NZ_BFCJ01000033.1	NZ_LPQA01000052.1	NC_019059.1	NC_010937.1	NC_014369.1
NZ_RXPP01000071.1	NZ_APWF01000060.1	NZ_BFCK01000011.1	NZ_LPQK01000015.1	NC_019060.1	NC_010980.1	NC_014959.1
NZ_RXPQ01000059.1	NZ_APWG01000101.1	NZ_BFCL01000054.1	NZ_LPQM01000037.1	NC_019061.1	NC_011101.1	NC_015054.1
NZ_RXPS01000070.1	NZ_APWJ01000039.1	NZ_BFCM01000058.1	NZ_LPQP01000039.1	NC_019070.1	NC_011102.1	NC_002123.1
NZ_RXQD01000071.1	NZ_AQQA01000132.1	NZ_BFCO01000016.1	NZ_LPQQ01000053.1	NC_019080.1	NC_011136.1	NC_010907.1
NC_019368.1	NZ_AQRD01000004.1	NZ_BFCQ01000022.1	NZ_LPQR01000048.1	NC_019081.1	NC_011139.1	NC_011073.1
NC_022652.1	NZ_ARPR01000194.1	NZ_BFCR01000070.1	NZ_LPQS01000029.1	NC_019082.1	NC_011140.1	NC_004771.1
CP016763.1	NZ_ARRU01000101.1	NZ_BFCS01000096.1	NZ_LPQW01000035.1	NC_019083.1	NC_011207.1	NC_011795.1
AF244145.1	NZ_ARRV01000080.1	NZ_BFCT01000116.1	NZ_LRJI01000043.1	NC_019085.1	NC_011228.1	NC_015950.1
AF445082.1	NZ_ARRZ01000061.1	NZ_BFCU01000014.1	NZ_LRJJ01000086.1	NC_019086.1	NC_011363.1	NC_015972.1
AY590475.1	NZ_ARSI01000101.1	NZ_BFCY01000147.1	NZ_LRJL01000082.1	NC_019087.1	NC_011364.1	NC_016009.1
AY795963.1	NZ_ARSJ01000057.1	NZ_BIFN01000180.1	NZ_LRJM01000044.1	NC_019088.1	NC_011378.1	NC_016039.1
DQ532122.1	NZ_ARSN01000117.1	NZ_BIFO01000144.1	NZ_LRJN01000051.1	NC_011514.1	NC_005013.1	NC_016053.1
JQ818252.1	NZ_ARSO01000220.1	NZ_CBYG010000041.1	NZ_LRJP01000075.1	NC_019092.1	NC_002517.1	NC_016600.1
JX101693.1	NZ_ARSP01000198.1	NZ_CCFB01000005.1	NZ_LXMI01000062.1	NC_019013.1	NC_002120.1	NC_016643.1
KF250428.1	NZ_ARSQ01000087.1	NZ_CDEL01000093.1	NZ_MCOM01000077.1	NC_019062.1	NC_001431.1	NC_016849.1
HQ419285.1	NZ_ARVM01000006.1	NZ_CDNA01000040.1	NZ_MCON01000196.1	NC_019065.1	NC_011405.1	NC_016852.1
KM977631.1	NZ_ARVP01000148.1	NZ_CDQG01000062.1	NZ_MCOO01000183.1	NC_019066.1	NC_011406.1	NC_016151.1
KF680003.1	NZ_ARVQ01000146.1	NZ_CGHE01000020.1	NZ_MCOP01000210.1	NC_019067.1	NC_011418.1	NC_016966.1

KT989376.1	NZ_ATAK01000080.1	NZ_CM001802.1	NZ_MCOQ01000136.1	NC_019068.1	NC_011497.1	NC_016973.1
KT982613.1	NZ_ATEZ01000138.1	NZ_CM008471.1	NZ_MDCW01000200.1	NC_019071.1	NC_006977.1	NC_016974.1
KT982618.1	NZ_AXLE01000003.1	NZ_CM008823.1	NZ_MDCX01000052.1	NC_019072.1	NC_011521.1	NC_016977.1
KT982615.1	NZ_AXLI01000004.1	NZ_CM008879.1	NZ_MDCY01000044.1	NC_019073.1	NC_011605.1	NC_016979.1
KT982615.1	NZ_AXLI01000005.1	NZ_CM008882.1	NZ_MDCZ01000168.1	NC_019074.1	NC_011610.1	NC_016976.1
KT982616.1	NZ_AZAP01000068.1	NZ_CM008884.1	NZ_MDDA01000130.1	NC_019075.1	NC_011617.1	NC_017213.1
KT982617.1	NZ_BFCA01000004.1	NZ_CM008885.1	NZ_MLFV01000055.1	NC_019076.1	NC_011640.1	NC_017197.1
KT989598.1	NZ_BFCN01000044.1	NZ_CM008891.1	NZ_MLFW01000070.1	NC_019077.1	NC_011642.1	NC_017489.1
KT989599.1	NZ_BFCP01000043.1	NZ_CM008895.1	NZ_MSAA01000028.1	NC_019079.1	NC_011752.1	NC_017478.1
KU051707.1	NZ_BFCX01000068.1	NZ_CM008904.1	NZ_NFFC01000079.1	NC_009131.1	NC_011754.1	NC_017772.1
KU051708.1	NZ_BFDC01000027.1	NZ_CM008917.1	NZ_NFFG01000090.1	NC_019093.1	NC_011799.1	NC_005705.1
KU051709.1	NZ_BFDD01000065.1	NZ_CM012324.1	NZ_NFFO01000062.1	NC_019094.1	NC_011812.1	NC_002799.1
KU051710.1	NZ_BFDE01000174.1	NZ_CM012324.1	NZ_NFFU01000013.1	NC_019095.1	NC_011964.1	NC_003101.1
KU886034.1	NZ_BFDF01000149.1	NZ_CP006661.1	NZ_NFFV01000038.1	NC_019096.1	NC_012006.1	NC_004058.1
KX711879.1	NZ_BFDG01000080.1	NZ_CP006799.1	NZ_NFFX01000040.1	NC_019097.1	NC_012031.1	NC_004772.1
KX711880.1	NZ_BGLK01000081.1	NZ_CP008933.1	NZ_NFFY01000029.1	NC_019056.1	NC_012208.1	NC_003201.1
KU726588.1	NZ_BGNU01000071.1	NZ_CP009115.1	NZ_NFFZ01000073.1	NC_019057.1	NC_012209.1	NC_004769.1
KU862632.1	NZ_BGNV01000079.1	NZ_CP010370.2	NZ_NFGB01000058.1	NC_019058.1	NC_012215.1	NC_004770.1
KY913900.1	NZ_BGNW01000137.1	NZ_CP010373.2	NZ_NFGC01000059.1	NC_019084.1	NC_012216.1	NC_008444.1
KY887590.1	NZ_BGNX01000052.1	NZ_CP010391.1	NZ_NFGM01000055.1	NC_019078.1	NC_012222.2	NC_008436.1
KY887595.1	NZ_BGNY01000121.1	NZ_CP010399.1	NZ_NFGN01000084.1	NC_009132.1	NC_012487.1	NC_008445.1
KY887596.1	NZ_BGNZ01000083.1	NZ_CP011839.1	NZ_NFGU01000072.1	NC_019139.1	NC_012547.1	NC_010290.1
KY887591.1	NZ_BGOA01000106.1	NZ_CP012754.1	NZ_NFGV01000057.1	NC_019140.1	NC_012625.1	NC_010291.1
CP021328.1	NZ_BGOB01000073.1	NZ_CP012901.1	NZ_NPIX01000118.1	NC_019141.1	NC_012626.1	NC_002798.1
CP022533.1	NZ_BGOC01000031.1	NZ_CP013483.1	NZ_NSMB01000103.1	NC_019142.1	NC_012627.1	NC_002150.1
CP024192.1	NZ_CCFQ01000037.1	NZ_CP014478.1	NZ_OOHS01000036.1	NC_019143.1	NC_012628.1	NC_005017.1
CP024522.1	NZ_CCFR01000024.1	NZ_CP014757.1	NZ_OOHT01000065.1	NC_019144.1	NC_012630.1	NC_005088.1
CP024529.1	NZ_CCFT01000028.1	NZ_CP015835.1	NZ_PDDP01000062.1	NC_019145.1	NC_012631.1	NC_013506.1
CP024557.1	NZ_CCGC01000012.1	NZ_CP016035.1	NZ_PDLU01000121.1	NC_019146.1	NC_012661.1	NC_016042.1
CP025964.2	NZ_CCGH01000106.1	NZ_CP016921.1	NZ_PEHD01000103.1	NC_019147.1	NC_012690.1	NC_016967.1
KY884003.1	NZ_CCHA01000013.1	NZ_CP017672.1	NZ_PUTD01000036.1	NC_019148.1	NC_012692.1	NC_017908.2
MF344557.1	NZ_CCHD01000041.1	NZ_CP018366.1	NZ_PUTE01000036.1	NC_019149.1	NC_012882.1	NC_010257.1
MF344559.1	NZ_CCHE01000047.1	NZ_CP018817.1	NZ_PUTF01000036.1	NC_019150.1	NC_012885.1	NC_005054.1
MF344561.1	NZ_CCHF01000083.1	NZ_CP019053.1	NZ_PXKU01000056.1	NC_018956.1	NC_003526.1	NC_001995.1
MF344562.1	NZ_CCHS01000075.1	NZ_CP020049.1	NZ_QDDT01000068.1	NC_018957.1	NC_006877.1	NC_005127.1
MF344564.1	NZ_CCHT01000060.1	NZ_CP020056.1	NZ_QDDU01000072.1	NC_018958.1	NC_005566.1	NC_005908.1
MF344565.1	NZ_CCHU01000057.1	NZ_CP020068.1	NZ_QDDW01000067.1	NC_006130.1	NC_006827.2	NC_013514.1
MF344567.1	NZ_CCHW01000023.1	NZ_CP020090.1	NZ_QDDX01000055.1	NC_004464.2	NC_005792.1	NC_002138.1
MG516907.1	NZ_CDFP01000006.1	NZ_CP020703.1	NZ_QFDS01000057.1	NC_006427.1	NC_002518.1	NC_017498.1
MG516908.1	NZ_CDFR01000233.1	NZ_CP020704.1	NZ_QKNQ01000003.1	NC_001370.1	NC_010286.1	NC_017500.1
MG516909.1	NZ_CM010658.1	NZ_CP020848.1	NZ_QKOX01000089.1	NC_002810.1	NC_010283.1	NC_013551.1
MG516910.1	NZ_CM010665.1	NZ_CP020854.1	NZ_QLNR01000077.1	NC_004965.1	NC_010279.1	NC_010281.1

MG516911.1	NZ_CP003997.1	NZ_CP020902.1	NZ_QWVW01000111.1	NC_000906.2	NC_010284.1	NC_010895.1
MG516912.1	NZ_CP007558.1	NZ_CP021206.1	NZ_QWWE01000104.1	NC_005010.1	NC_010722.1	NC_004443.1
MF072962.1	NZ_CP007732.1	NZ_CP021345.1	NZ_RDWT01000101.1	NC_004562.1	NC_007926.1	NC_010112.1
CP027603.1	NZ_CP008791.1	NZ_CP021345.1	NZ_RYXN01000077.1	NC_002637.1	NC_012944.1	NC_002108.1
CP028952.1	NZ_CP008798.1	NZ_CP021536.1	NZ_UISC01000060.1	NC_005565.1	NC_013175.1	NC_016601.1
CP028486.1	NZ_CP008824.1	NZ_CP021551.1	NZ_UISE01000058.1	NC_006976.1	NC_002192.1	NC_001372.1
CP031121.1	NZ_CP008833.1	NZ_CP021687.1	NZ_UISK01000113.1	NC_002092.1	NC_002610.1	NC_009444.1
MH727565.1	NZ_CP008901.1	NZ_CP021699.1	NZ_UITI01000068.1	NC_002130.1	NC_004846.1	NC_013128.1
MH882484.1	NZ_CP008901.1	NZ_CP021709.1	NZ_UIVF01000087.1	NC_006997.1	NC_004968.1	NC_013319.1
MH829594.1	NZ_CP009853.1	NZ_CP021720.1	NZ_UIXR01000051.1	NC_005923.1	NC_006881.1	NC_013320.1
AB636651.1	NZ_CP009858.1	NZ_CP021936.1	NZ_UIXU01000060.1	NC_005324.1	NC_008443.1	NC_013289.1
LC107424.1	NZ_CP009862.1	NZ_CP021941.1	NZ_UJAY01000132.1	NC_004958.1	NC_008612.1	NC_013372.1
LC107606.1	NZ_CP009867.1	NZ_CP021947.1	NZ_UJHZ01000086.1	NC_006994.1	NC_011641.1	NC_013387.1
LC198842.1	NZ_CP009875.1	NZ_CP021952.1	NZ_UJIF01000084.1	NC_002487.1	NC_011796.1	NC_018952.1
AJ609296.3	NZ_CP009876.1	NZ_CP021961.1	NZ_UJKO01000087.1	NC_004652.1	NC_012886.1	NC_013338.1
KQ089505.1	NZ_CP009881.1	NZ_CP021962.1	NZ_UJME01000055.1	NC_002119.1	NC_002129.1	NC_013339.1
NIII01000041.1	NZ_CP010362.1	NZ_CP022126.1	NZ_UJML01000088.1	NC_001537.1	NC_002111.1	NC_013309.1
NIIR01000049.1	NZ_CP010374.1	NZ_CP022350.1	NZ_UJWL01000026.1	NC_004944.1	NC_007207.1	NC_013311.1
NPGF01000059.1	NZ_CP010379.1	NZ_CP022612.1	NZ_UJWM01000030.1	NC_005098.1	NC_007209.1	NC_013375.2
NGAG01000014.1	NZ_CP010396.1	NZ_CP023187.1	NZ_UJWN01000027.1	NC_005322.1	NC_004940.1	NC_013350.1
NGAL01000047.1	NZ_CP010575.1	NZ_CP023274.1	NZ_UJWQ01000026.1	NC_006278.1	NC_004747.1	NC_013351.1
NGAJ01000013.1	NZ_CP011571.1	NZ_CP023488.1	NZ_UJXD01000027.1	NC_002145.1	NC_005323.1	NC_013352.1
PCFT01000003.1	NZ_CP011573.1	NZ_CP023554.1	NZ_UKNV01000033.1	NC_005019.1	NC_005248.1	NC_013314.1
RDRD01000120.1	NZ_CP011580.1	NZ_CP023910.1	NZ_UKRC01000036.1	NC_000937.1	NC_009841.1	NC_013388.1
RXQD01000071.1	NZ_CP011582.1	NZ_CP023914.1	NZ_UKXI01000027.1	NC_000938.1	NC_004768.1	NC_013322.1
RXPP01000071.1	NZ_CP011595.1	NZ_CP023923.1	NZ_UKXU01000032.1	NC_001949.1	NC_004978.1	NC_013373.1
RXPS01000070.1	NZ_CP011598.1	NZ_CP023948.1	NZ_UKXU01000032.1	NC_004960.1	NC_010428.1	NC_013323.1
	NZ_CP011608.1	NZ_CP024039.1	NZ_ULAU01000072.1	NC_003893.1	NC_010419.1	NC_013326.1
	NZ_CP011611.1	NZ_CP024814.1	NZ_ULAA01000085.1	NC_003894.1	NC_010423.2	NC_018972.1
	NZ_CP011615.1	NZ_CP024828.1	NZ_ULAU01000072.1	NC_009034.1	NC_010796.1	NC_013329.1
	NZ_CP011622.1	NZ_CP024879.1	NZ_ULAU01000072.1	NC_009353.1	NC_010880.1	NC_013374.1
	NZ_CP011635.1	NZ_CP026127.1	NZ_ULAW01000053.1	NC_009739.1	NC_010881.1	NC_013336.1
	NZ_CP011639.1	NZ_CP026127.1	NZ_ULDL01000044.1	NC_005327.1	NC_010883.1	NC_013303.1
	NZ_CP011649.1	NZ_CP026127.1	NZ_ULDM01000044.1	NC_003350.1	NC_010901.1	NC_013304.1
	NZ_CP011656.1	NZ_CP026590.1	NZ_ULDP01000090.1	NC_004999.1	NC_010904.1	NC_013343.1
	NZ_CP011661.1	NZ_CP026617.1	NZ_ULDS01000088.1	NC_007365.1	NC_002630.1	NC_013317.1
	NZ_CP011981.1	NZ_CP027177.1	NZ_ULEA01000084.1	NC_005249.1	NC_013120.1	NC_013347.1
	NZ_CP011986.1	NZ_CP027528.1	NZ_UWWA01000004.1	NC_006323.1	NC_013122.1	NC_013348.1
	NZ_CP011991.1	NZ_CP027532.1	NZ_UWXE01000002.1	NC_008275.1	NC_013176.1	NC_013349.1
	NZ_CP013325.1	NZ_CP027679.1	NC_014368.1	NC_002525.1	NC_013277.1	NC_018979.1
	NZ_CP015347.1	NZ_CP028171.1	NC_019375.1	NC_005211.1	NC_013503.1	NC_018980.1
	NZ_CP015382.1	NZ_CP028178.1	NC_021576.1	NC_005205.1	NC_013507.1	NC_018989.1
	NZ_CP015395.1	NZ_CP028560.1	NC_021622.1	NC_004564.1	NC_013533.1	NC_018990.1

	NZ_CP015990.1	NZ_CP028786.2	NC_022242.1	NC_005024.1	NC_013541.1	NC_018991.1
	NZ_CP015991.1	NZ_CP028800.1	NC_024987.1	NC_005250.1	NC_013542.1	NC_018992.1
	NZ_CP016381.1	NZ_CP028929.1	NC_025186.1	NC_008357.1	NC_013543.1	NC_018993.1
	NZ_CP017083.1	NZ_CP029118.1	NC_032101.1	NC_006671.1	NC_013544.1	NC_018994.1
	NZ_CP017086.1	NZ_CP029397.1	AF317511.1	NC_008460.1	NC_013545.1	NC_018995.1
	NZ_CP017929.1	NZ_CP029610.1	AY152821.1	NC_008490.1	NC_013549.1	NC_018996.1
	NZ_CP017937.1	NZ_CP029631.1	AY339625.2	NC_008613.1	NC_013550.1	NC_019000.1
	NZ_CP017993.1	NZ_CP029731.1	AY648125.1	NC_006277.2	NC_013589.1	NC_013331.1
	NZ_CP018356.1	NZ_CP029737.1	AY970968.1	NC_002377.1	NC_013652.1	NC_019003.1
	NZ_CP018362.1	NZ_CP030345.1	AY987853.1	NC_001911.1	NC_013653.1	NC_019004.1
	NZ_CP018426.1	NZ_CP030858.1	DQ112355.1	NC_009139.1	NC_013728.1	NC_019006.1
	NZ_CP018432.1	NZ_CP031216.1	DQ489717.1	NC_009435.1	NC_013780.1	NC_013390.1
	NZ_CP018436.1	NZ_CP031297.1	DQ522239.1	NC_004998.1	NC_013782.1	NC_013394.1
	NZ_CP018455.1	NZ_CP031322.1	EF078697.1	NC_007675.1	NC_013783.1	NC_013384.1
	NZ_CP018668.1	NZ_CP031716.1	EF093145.1	NC_009602.1	NC_013789.1	NC_013371.1
	NZ_CP018669.1	NZ_CP031736.1	EF093146.1	NC_010076.1	NC_013950.1	NC_013318.1
	NZ_CP018884.1	NZ_CP031884.1	EF494743.1	NC_010111.1	NC_013969.1	NC_013324.1
	NZ_CP018887.1	NZ_CP032132.1	EF577408.1	NC_007772.1	NC_014105.1	NC_013327.1
	NZ_CP018945.1	NZ_CP032142.1	EF690695.1	NC_006828.1	NC_014111.1	NC_013335.1
	NZ_CP018949.1	NZ_CP032193.1	EF690696.1	NC_006829.1	NC_014113.1	NC_013337.1
	NZ_CP018956.1	NZ_CP032278.1	EU118150.1	NC_007928.1	NC_014124.1	NC_013302.1
	NZ_CP018959.1	NZ_CP032284.1	FJ172675.1	NC_007931.1	NC_014156.1	NC_019011.1
	NZ_CP018963.1	NZ_CP032284.1	GQ422826.1	NC_005018.1	NC_014208.1	NC_019018.1
	NZ_CP018968.1	NZ_CP032286.1	GQ422827.1	NC_002144.1	NC_014234.1	NC_013325.1
	NZ_CP018974.1	NZ_CP033133.1	GQ422828.1	NC_002096.1	NC_014312.1	NC_013377.1
	NZ_CP018977.1	NZ_CP033439.1	GQ422829.1	NC_005020.1	NC_014356.1	NC_013378.1
	NZ_CP018989.1	NZ_CP034323.1	GQ161847.1	NC_002635.1	NC_014367.1	NC_013380.1
	NZ_CP018999.1	NZ_CP034369.1	GU724868.1	NC_006843.1	NC_014368.1	NC_013381.1
	NZ_CP019001.1	NZ_CP034398.1	GU724869.1	NC_001767.1	NC_014382.1	NC_013382.1
	NZ_CP019006.1	NZ_CP034403.1	GU724870.1	NC_005015.1	NC_014383.1	NC_013389.1
	NZ_CP019017.1	NZ_CP034409.1	GU724871.1	NC_005567.1	NC_014385.1	NC_013294.1
	NZ_CP019026.1	NZ_CP035739.1	GU724872.1	NC_001277.1	NC_014475.1	NC_013295.1
	NZ_CP020110.1	NZ_CTBD01000091.1	GU585907.1	NC_007100.1	NC_014477.1	NC_013296.1
	NZ_CP021328.1	NZ_CVLD01000041.1	HM126016.1	NC_008356.1	NC_014478.1	NC_013298.1
	NZ_CP021743.1	NZ_CVLE01000125.1	JQ690541.1	NC_008487.1	NC_014496.1	NC_013332.1
	NZ_CP021750.1	NZ_CVLT01000087.1	JQ824049.1	NC_008488.1	NC_014615.1	NC_013333.1
	NZ_CP021835.1	NZ_CZWB01000057.1	CP003739.1	NC_008489.1	NC_014627.1	NC_013334.1
	NZ_CP021853.1	NZ_FKLP01000165.1	KC140564.1	NC_004981.2	NC_014235.1	NC_013306.1
	NZ_CP022275.1	NZ_FLXD01000026.1	KC417377.1	NC_008597.1	NC_014726.1	NC_013330.1
	NZ_CP022276.1	NZ_FLXG01000027.1	KC417378.1	NC_004988.1	NC_014813.1	NC_019007.1
	NZ_CP022349.1	NZ_FNXW01000145.1	KC417379.1	NC_005243.1	NC_014843.1	NC_013297.1
	NZ_CP022574.1	NZ_FWFB01000125.1	KC430094.1	NC_005564.1	NC_015055.1	NC_013310.1

	NZ_CP022693.1	NZ_FWWO01000105.1	JX424614.1	NC_002757.1	NC_015154.1	NC_019010.1
	NZ_CP022698.1	NZ_FXMF01000024.1	KF856617.1	NC_005000.1	NC_015171.1	NC_013376.2
	NZ_CP022917.1	NZ_FXMG01000023.1	KF850516.1	NC_002013.1	NC_015175.1	NC_013321.1
	NZ_CP022920.1	NZ_FXMP01000036.1	KF977034.1	NC_008768.1	NC_015256.1	NC_013290.1
	NZ_CP022923.1	NZ_FXNB01000025.1	KJ547703.1	NC_010113.1	NC_015432.1	NC_013291.1
	NZ_CP022926.1	NZ_FXNE01000025.1	KF998104.1	NC_010114.1	NC_015472.1	NC_013328.1
	NZ_CP023186.1	NZ_FXNP01000028.1	KM514484.1	NC_010177.2	NC_015599.1	NC_018977.1
		NZ_FXOE01000054.1	KM975294.1	NC_003265.1	NC_004843.1	NC_018978.1
				NC_010230.1	NC_005246.1	NC_018981.1

5 PCA

Below are the 0% error rate PCAs for all BOC reads for the genomes, individual plasmids and group plasmids.

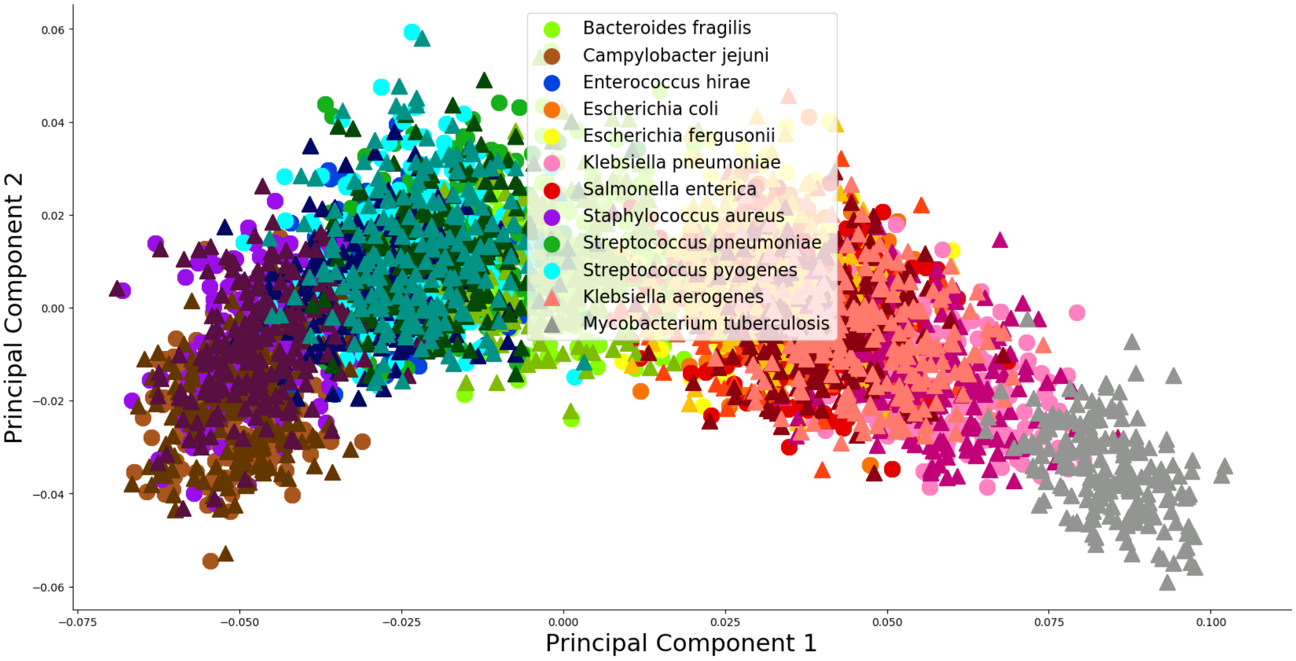

Supplemental Figure 5. PCA for genomes at $m = 0$ and $r = 10^2$. Circles represent the training genome and triangles represent the testing genomes.

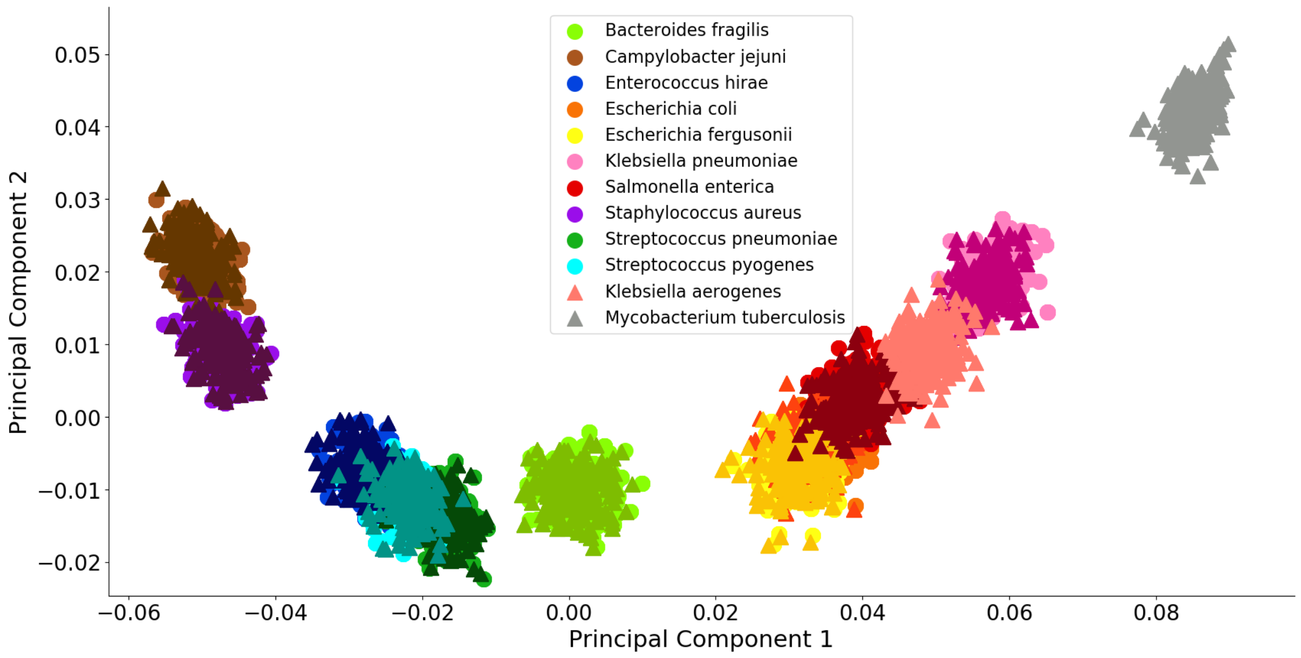

Supplemental Figure 6. PCA for genomes at $m = 0$ and $r = 10^3$. Circles represent the training genome and triangles represent the testing genomes.

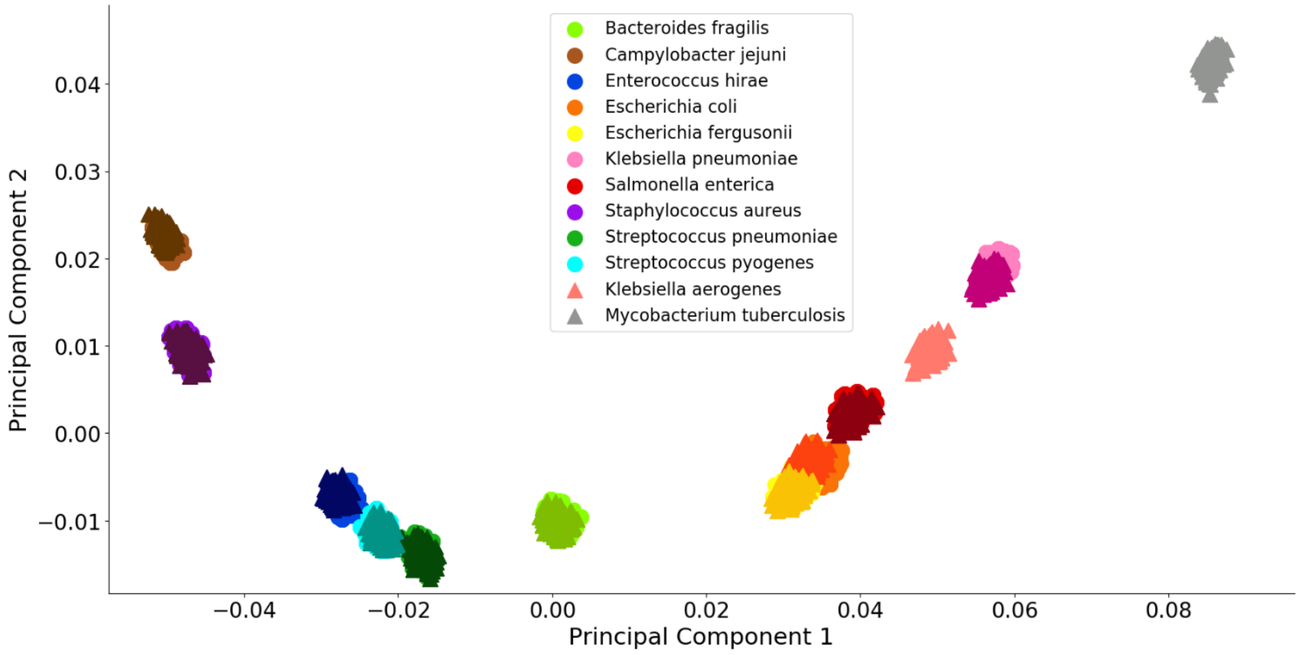

Supplemental Figure 7. PCA for genomes at $m = 0$ and $r = 10^4$. Circles represent the training genome and triangles represent the testing genomes.

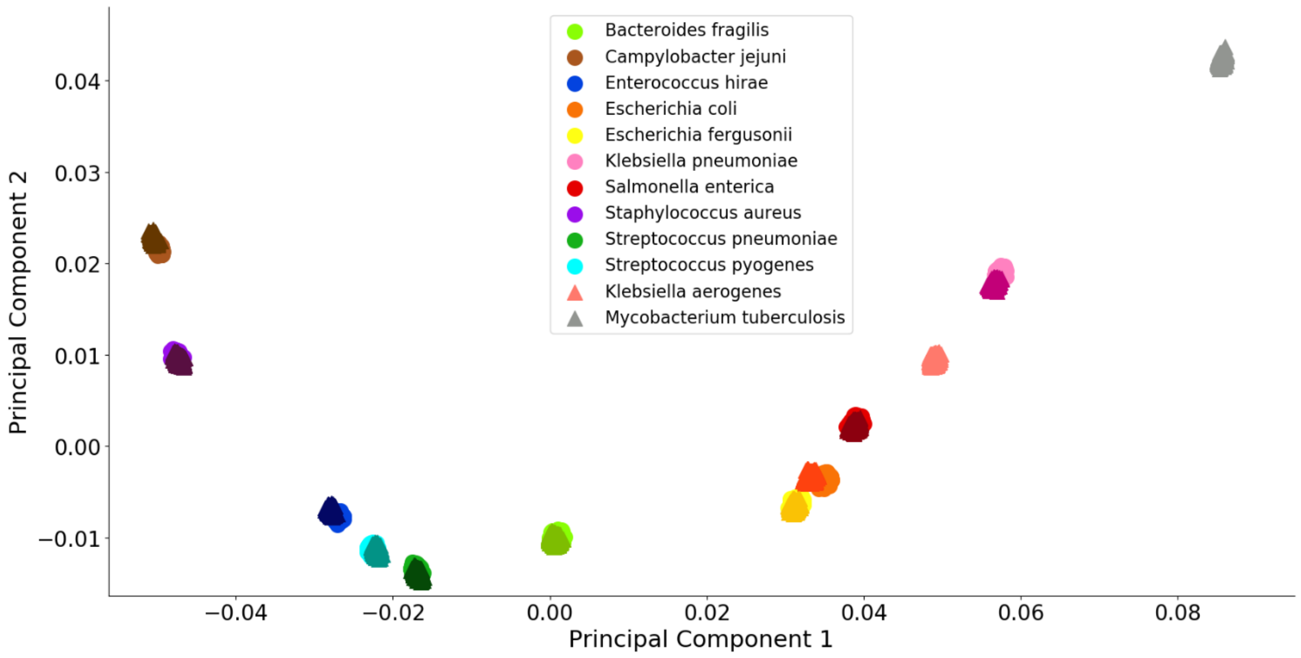

Supplemental Figure 8. PCA for genomes at $m = 0$ and $r = 10^5$. Circles represent the training genome and triangles represent the testing genomes.

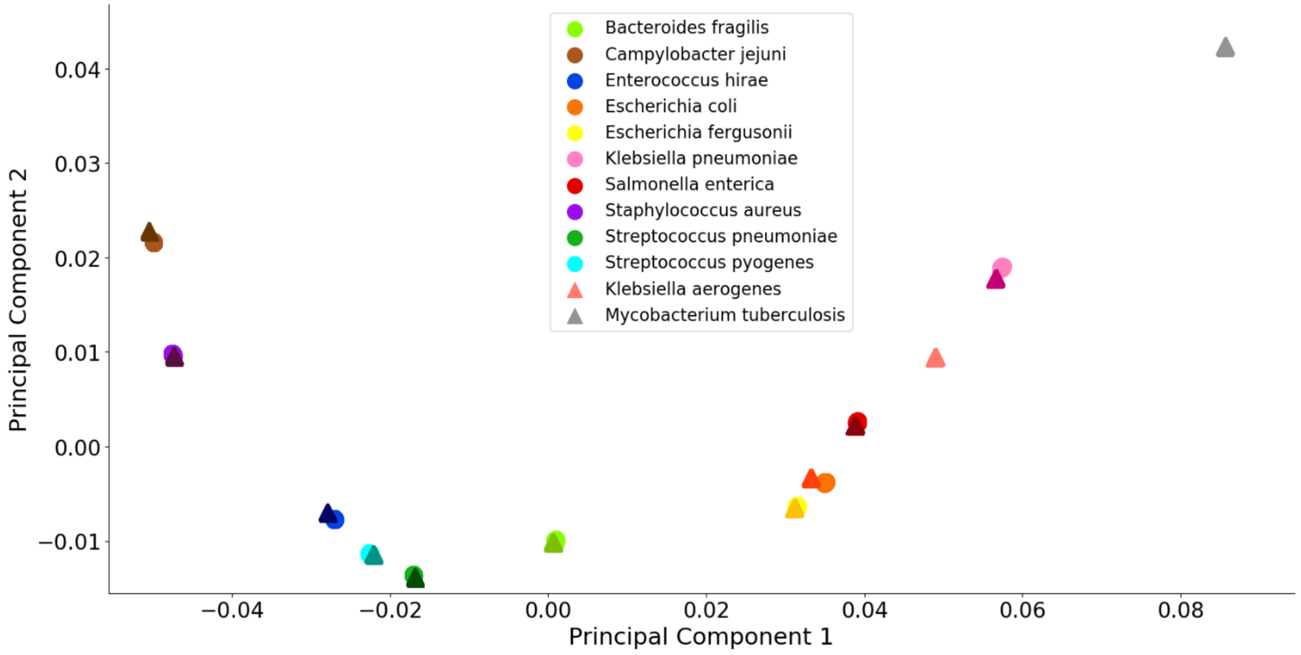

Supplemental Figure 9. PCA for genomes at $m = 0$ and $r = 10^6$. Circles represent the training genome and triangles represent the testing genomes.

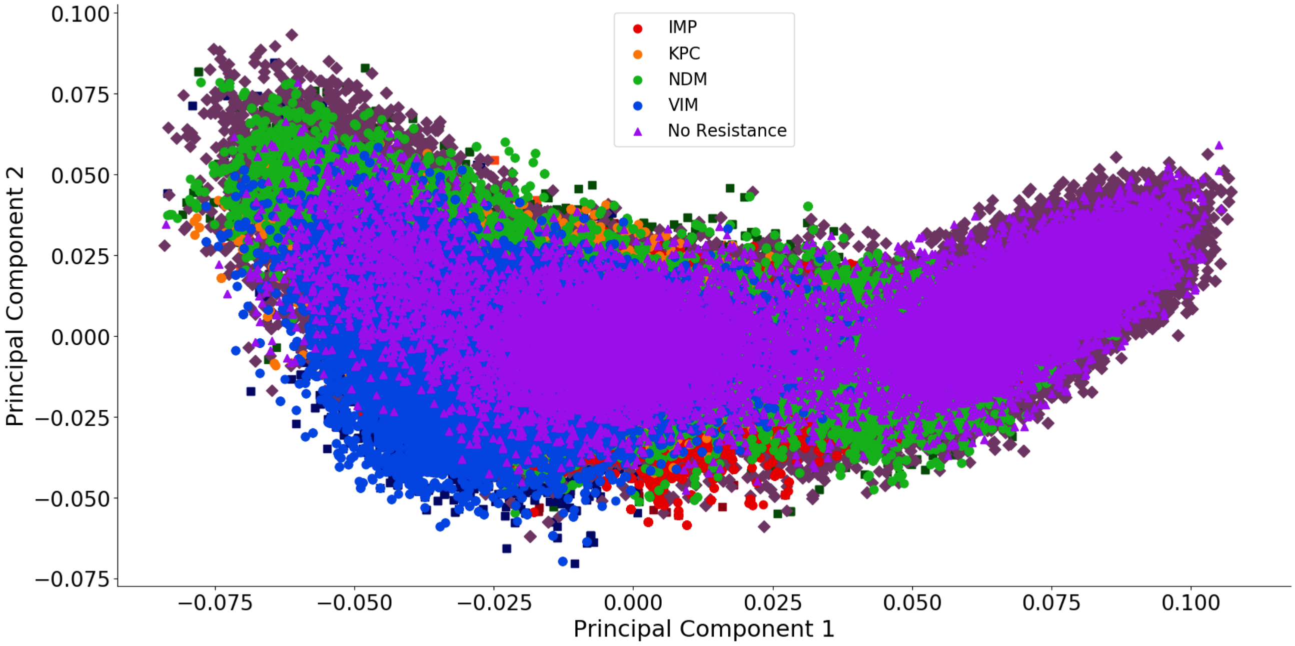

Supplemental Figure 10. PCA for individual plasmid identification at $m = 0$ and $r = 10^2$. Circles represent the training plasmids and triangles represent the testing plasmids.

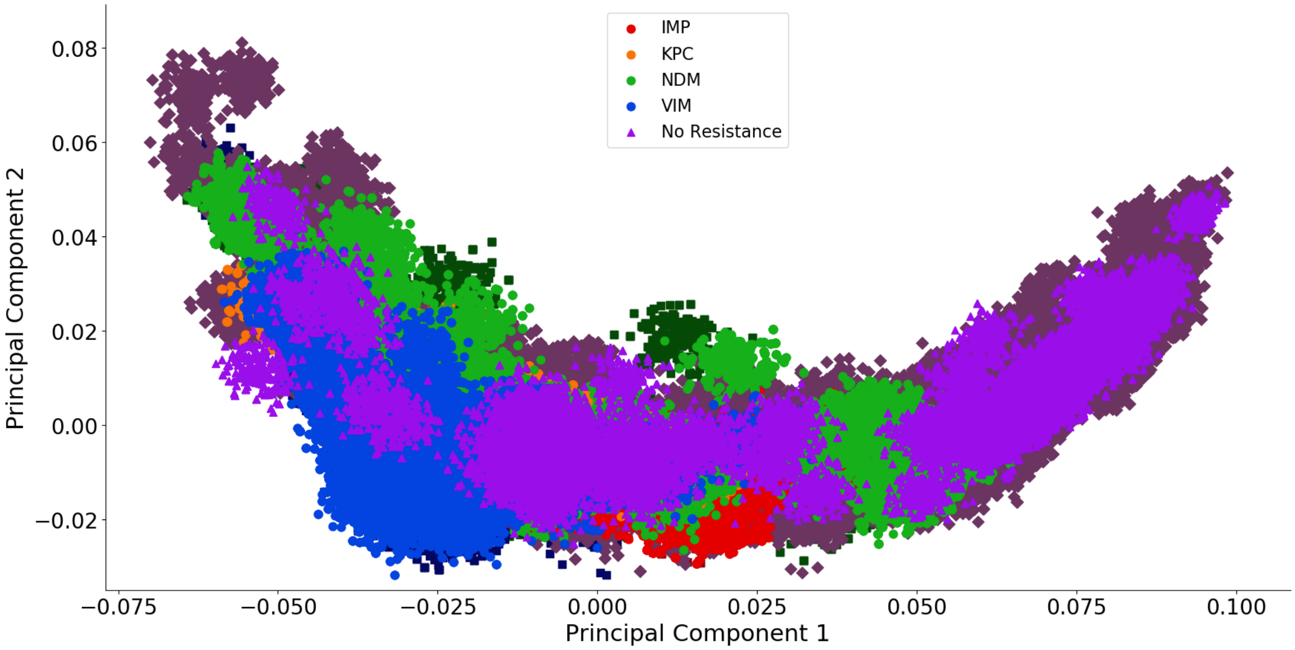

Supplemental Figure 11. PCA for individual plasmid identification at $m = 0$ and $r = 10^3$. Circles represent the training plasmids and triangles represent the testing plasmids.

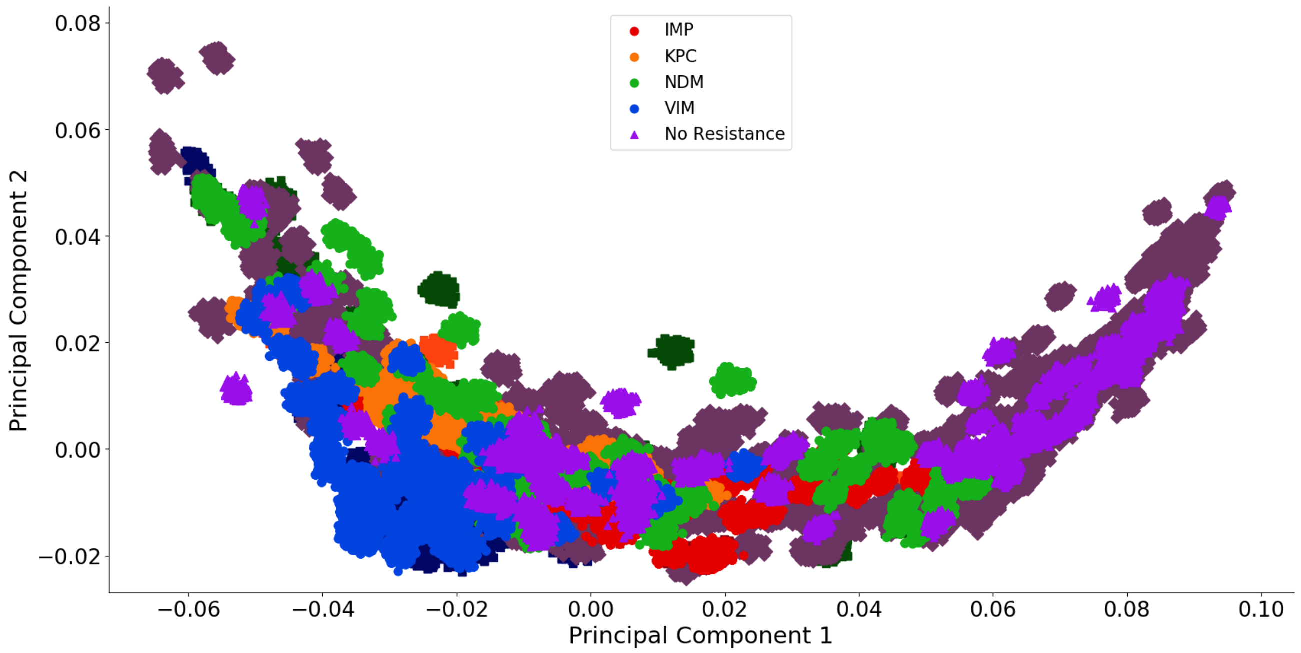

Supplemental Figure 12. PCA for individual plasmid identification at $m = 0$ and $r = 10^4$. Circles represent the training plasmids and triangles represent the testing plasmids.

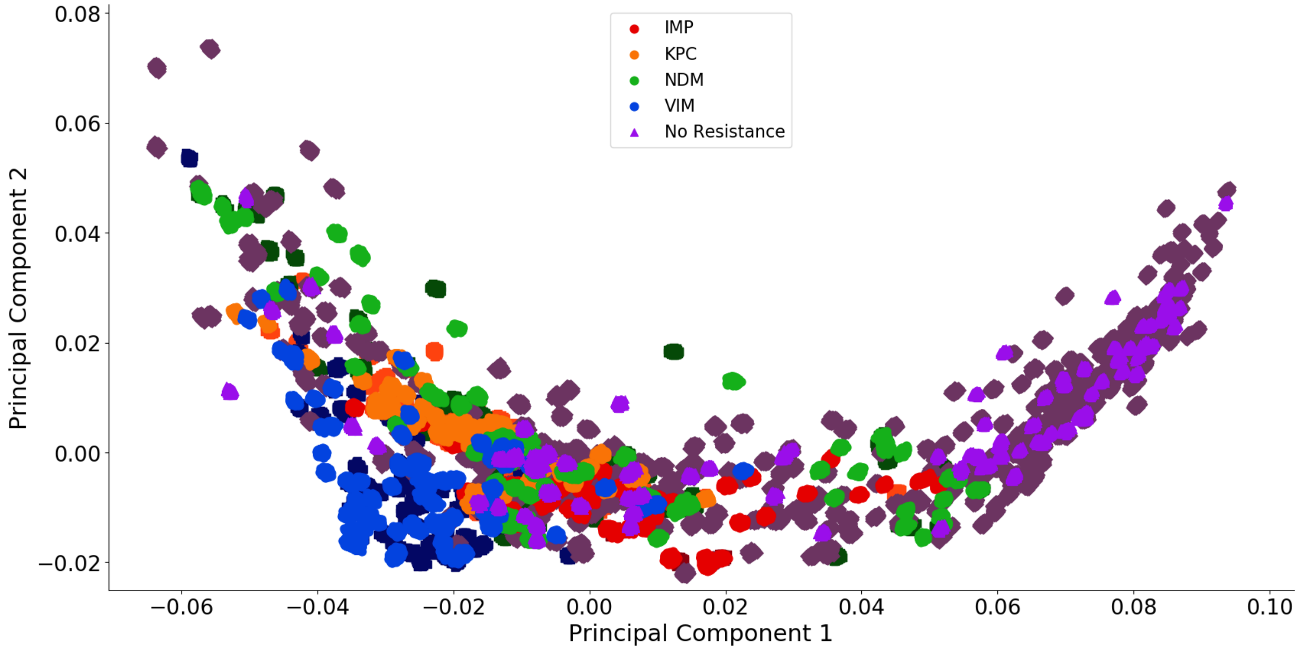

Supplemental Figure 13. PCA for individual plasmid identification at $m = 0$ and $r = 10^5$. Circles represent the training plasmids and triangles represent the testing plasmids.

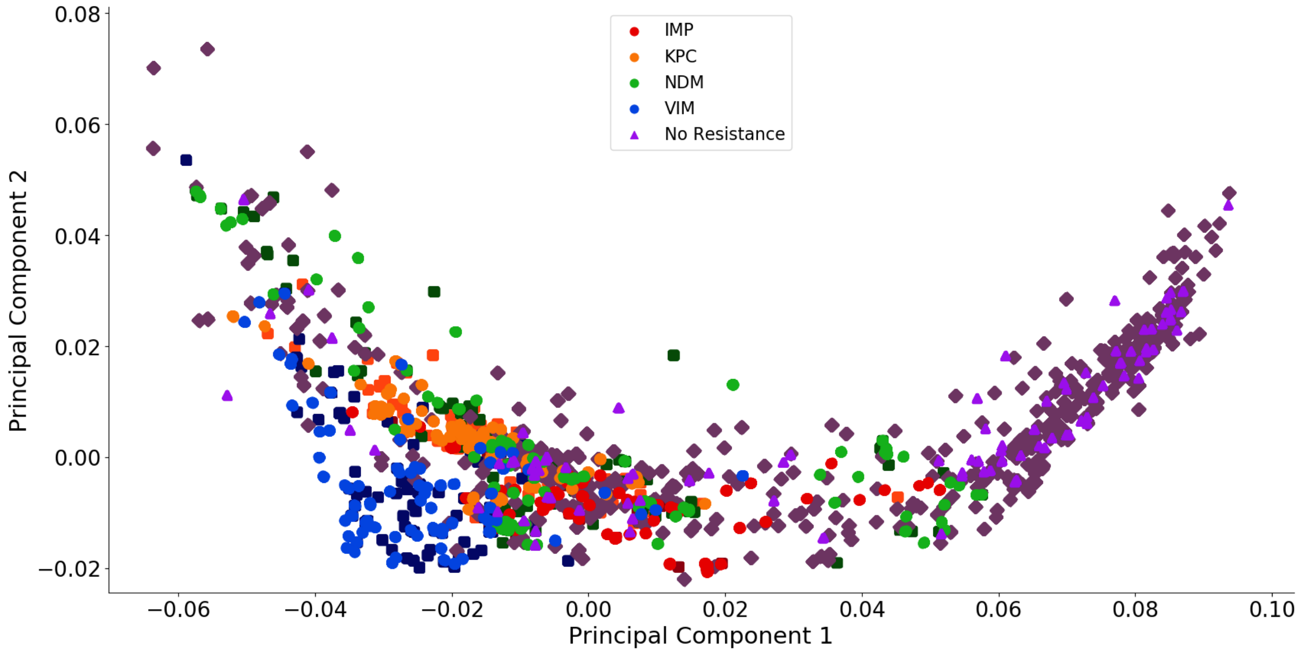

Supplemental Figure 14. PCA for individual plasmid identification at $m = 0$ and $r = 10^6$. Circles represent the training plasmids and triangles represent the testing plasmids.

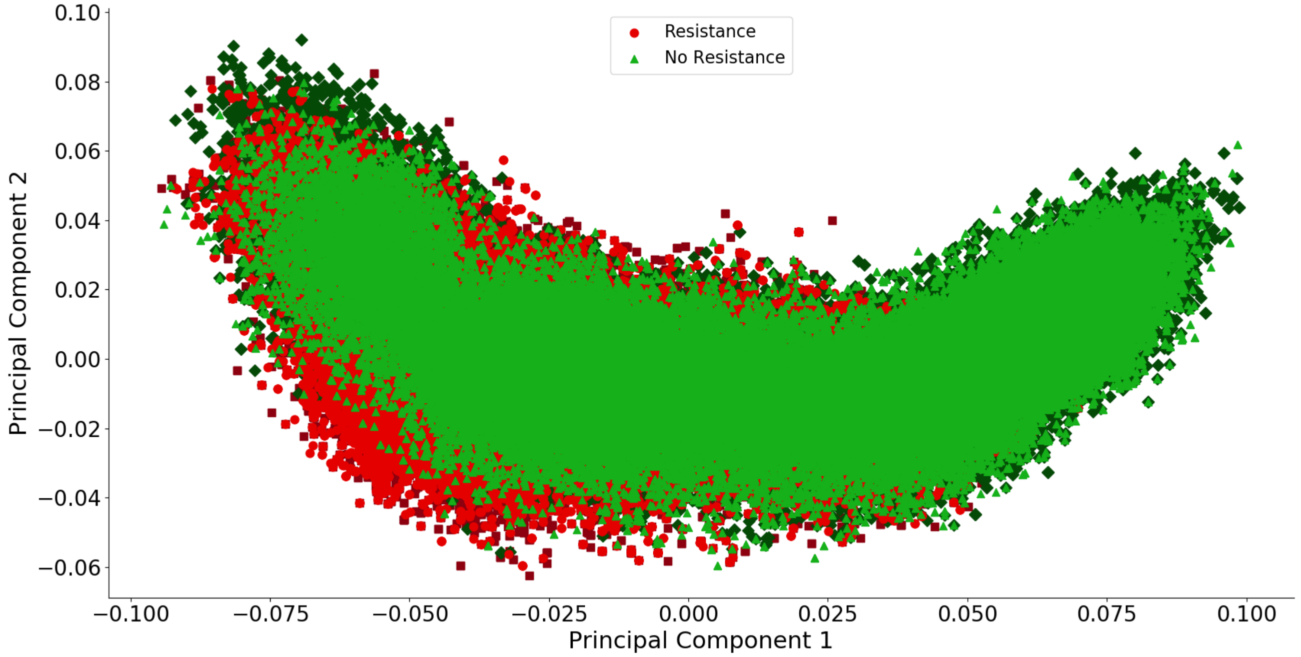

Supplemental Figure 15. PCA for group plasmid identification at $m = 0$ and $r = 10^2$. Circles represent the training plasmids and triangles represent the testing plasmids.

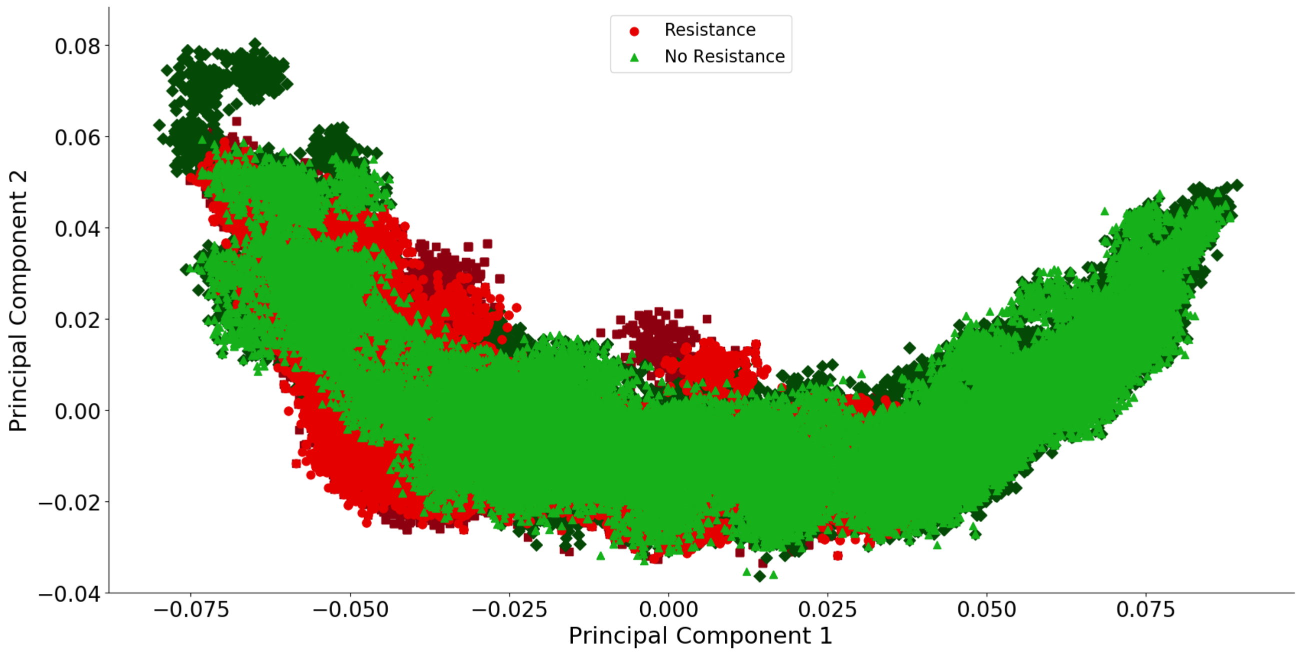

Supplemental Figure 16. PCA for group plasmid identification at $m = 0$ and $r = 10^3$. Circles represent the training plasmids and triangles represent the testing plasmids.

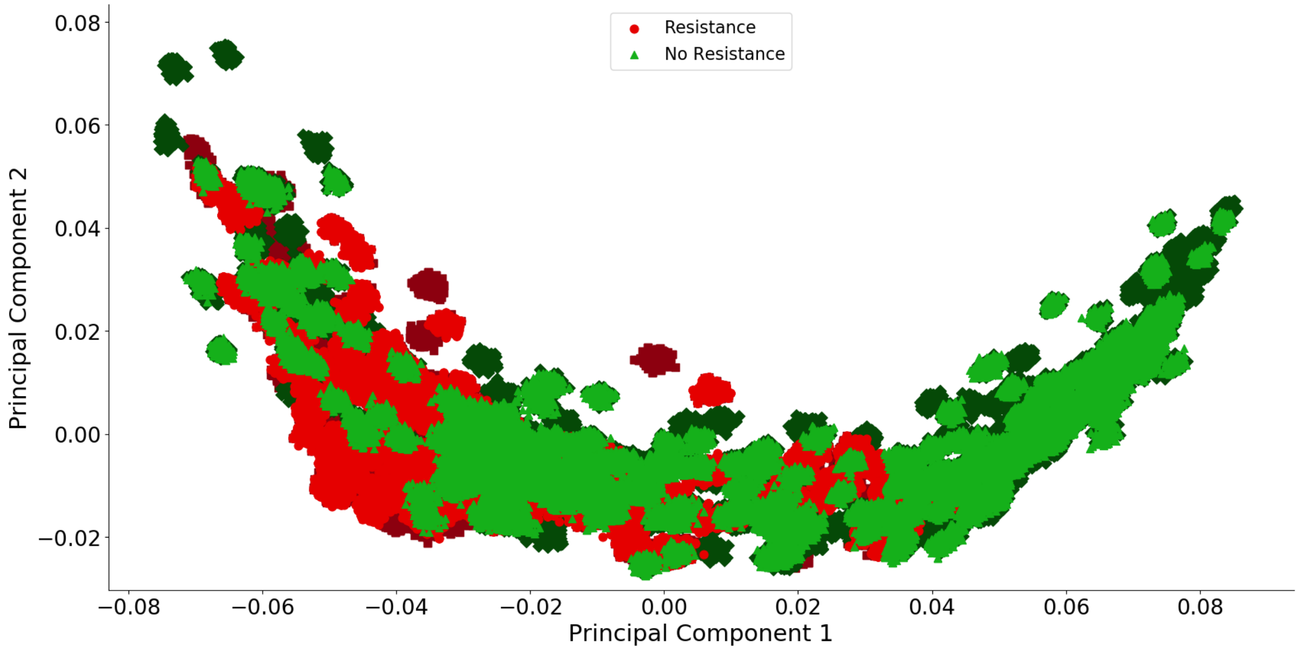

Supplemental Figure 17. PCA for group plasmid identification at $m = 0$ and $r = 10^4$. Circles represent the training plasmids and triangles represent the testing plasmids.

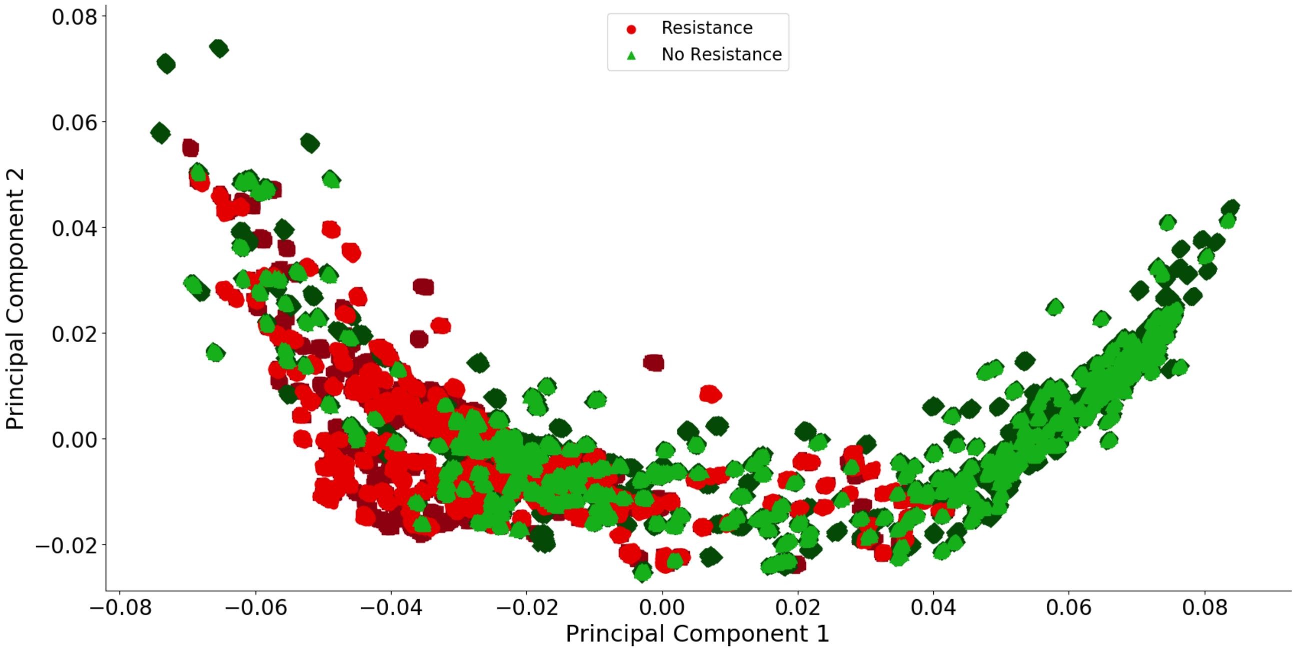

Supplemental Figure 18. PCA for group plasmid identification at $m = 0$ and $r = 10^5$. Circles represent the training plasmids and triangles represent the testing plasmids.

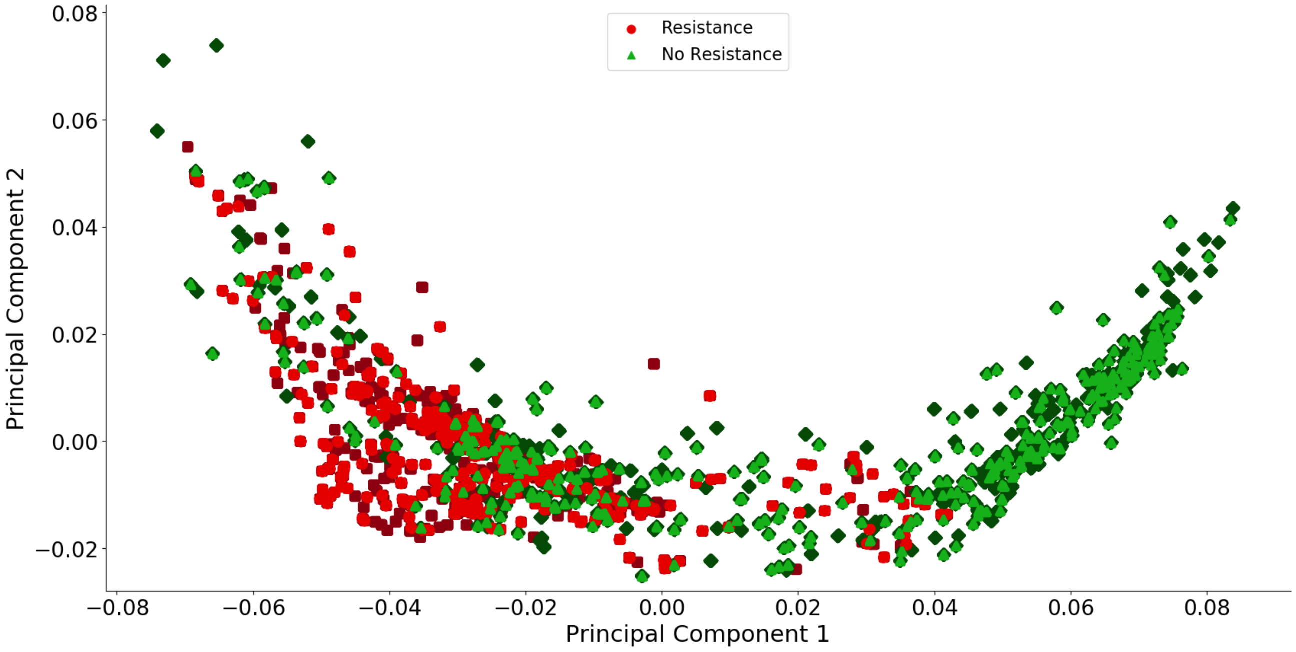

Supplemental Figure 19. PCA for group plasmid identification at $m = 0$ and $r = 10^6$. Circles represent the training plasmids and triangles represent the testing plasmids.
